# Effect of cerium oxide on physical, structural, and spectroscopic properties of tellurium-borate glasses for cool greenish light emitting devices

**DOI:** 10.1038/s41598-026-40883-y

**Published:** 2026-02-19

**Authors:** B. N. Shiva Kumar, D. Vinay, C. Devaraja

**Affiliations:** Department of Physics, Manipal Institute of Technology Bengaluru, Manipal Academy of Higher Education, Manipal, 576104 Karnataka India

**Keywords:** Tellurium soda-borate glasses, Cerium Oxide, Oxygen packing density, Metallization criterion, Linear dielectric susceptibility, Materials science, Optics and photonics, Physics

## Abstract

This investigation is to explore how CeO_2_ affects the physical, structural, and spectroscopic characteristics of tellurium soda-borate glasses for potential applications in optoelectronics. The conventional melt quenching technique was used to produce the necessary glasses by adding different amounts of CeO_2_ to the components of the tellurium soda-borate glasses. The non-crystalline nature of the samples was validated by the X-ray diffraction patterns. The density of glass samples was determined by Archimedes principle, and hence other physical properties like polaron radius, oxygen packing density, and average boron-boron separation were calculated. The FTIR spectra demonstrated the presence of fundamental structural groups of borate (BO_3_ and BO_4_) and tellurite (TeO_4_ and TeO_3_) in both the undoped and doped samples. FTIR also demonstrated the presence of the unique structural group of cerium tetrahedral CeO_4_ when the B_2_O_3_ level was decreased. The optical properties were analysed for the glass samples by the UV-Visible spectroscopy method. Refractive index (RI) of the glasses was found by using suitable mathematical approaches, and the highest RI value was found for BTNC5. Metallization criterion, optical basicity, electronegativity, and optical properties are determined with the help of the obtained RI and optical energy bandgap. The values of direct and indirect energy gaps, optical basicity, and refractive index were all impacted by the variation of CeO_2_ content. The CeO_2_-doped BTNC glasses allowed 5d → 4f transitions of Ce^3+^ ions and gave a wide green emission at 512 nm. The CIE diagram showed that CeO_2_-doped BTNC glasses lie in the green to yellowish region. The CCT values are > 5000 K, indicating a cool CCT. The obtained results of optical and luminescence properties indicate that the BTNC glasses are potential candidates for light-emitting devices.

## Introduction

In recent years, many researchers have been working on metal oxide glasses due to their promising applications in the field of radiation protection, industrial facilities, optical switching, optoelectronic devices, fibre optics, and medical diagnostics procedures like X-ray diffraction^1–7^. Because of exceptional physics and chemical properties such as ease of synthesis, low melting point, good thermal stability, low cost, relatively high chemical durability, and high refractive indices, metal oxide glasses have garnered greater interest^8–12^. B_2_O_3_ is one of the most significant glasses among others, which can be added as a flux material to various types of glass systems to create materials with enriched physical and chemical properties for appropriate advanced technological applications. Mostly, the borate in crystals and glasses produces basic structural units signified by [BO_3_] and [BO_4_], coordinating with three or four oxygen atoms. Subsequently, pure B_2_O_3_ glass is known to be generally composed of boroxol ring B_3_O_9/2_ with three-coordinate BO_3_ units; hence, to avoid the formation of boroxol rings in glass, it is required to add alkali metals to change the structure of the glass^13–18^.

Tellurite has attracted significant attention due to its exceptional characteristics, which put it apart from phosphate and silicate glasses. These unique properties include a higher linear refractive index (> 2), good thermal stability (ΔT > 100 °C), wide optical transmission (350 nm − 5 *µ*m), low phonon energy, and low melting temperature (700 °C for TeO_2_ glassy materials)^19–24^. These properties are highly suitable for applications in optical fibres^21,22^, amplifiers^25,26^, sensors^20^, and laser technology^25,26^. Additionally, it is well known that TeO_2_ pure glass requires a high cooling rate. But this can be achieved only under precise conditions; hence, to synthesise the tellurite glasses by using the melt quenching process, alkali oxides such as Li_2_O, Na_2_O, and K_2_O, etc., need to be added to the glass network to assist in vitrification^27^. Rare earth oxides (REOs) are preferably added to the glass matrix to boost the physical, optical, and luminescent properties^3,5,6,28,29^. In addition, the density is the most useful feature since it assists with phosphor, luminescent, and gamma-ray shielding materials^9,12,30,31^. Glasses containing REOs are found as potential candidates for optical fibers, lasers, scintillation detectors, and medical picture display^2,3,5,6,8,32^. The low toxicity Ce ions doped host materials exhibit valence states 4 (Ce^4+^) and 3 (Ce^3+^). Ce^3+^ (^4^f_1_) gives transitions between 4f and 5 d electronic structures, which are permitted by parity. Additionally, Ce^4+^, which is added to the glass network, will trap electrons and give no luminescence, but absorption characteristics related to charge transfer. At room temperature, the Ce^4+^ shows a broad absorption spectrum due to its charge transfer bond in the UV region where whereas Ce^3+^ ions absorb at 320 nm and emit at 450 nm with a decay time of 120 ns^33–35^ S. R. Rejishal et al.^36^ reported the effect of cerium (IV) oxide on the optical and dielectric properties of strontium bismuth borate glasses and, described the physical, optical, structural, and dielectric properties of 20SrO – (15-x) Bi_2_O_3_ – xCeO_2_ − 65B_2_O_3_ (0 ≤ x ≤ 5, mol%) glasses and also reported that the glasses prepared in the reducing synthesis environment exhibits a significant improvement in Ce^3+^ luminescence, and indicates the Ce^4+^ ions were effectively reduced to the Ce^3+^ state. Nevertheless, the development in the structural, optical, and physical properties of CeO_2_-doped boro tellurite glasses is still essential to discover more by varying the proper glass composition. E. Ibrahim et al.^37^ investigated the structural, optical, and dielectric properties of tellurite borate glasses doped with cerium oxide.

The current work involves the synthesis of sodium boro-tellurite glasses doped with CeO_2_: B_2_O_3_-TeO_2_-Na_2_O-CeO_2_. The focus of this work is to investigate the physical, structural, optical, and luminescence properties of the sodium boro-tellurite glasses doped with CeO_2_, and to check the suitability of these glasses for applications in optical materials, optical fibres, and optical switching applications.

## Materials and methods

A new set of glass samples having chemical formula (65-y)B_2_O_3_−25TeO_2_−10Na_2_O-yCeO_2_ (where y = 0, 0.1, 0.2, 0.3, 0.4, and 0.5 mol %) have been synthesized by the melt quenching method. The essential chemicals with analytical grade, such as H_3_BO_3_ (99% purity by Merch), Na_2_CO_3_ (99.9% purity from Sigma Aldrich), TeO_2_ (99.9% purity from Loba), and Cerium oxide CeO_2_ (99.9% purity from Sigma Aldrich), have been taken as starting materials for synthesis. Before placing these chemicals into the porcelain crucibles, the chemicals were grinded/stirred for one hour in a mortar and pestle to acquire a homogeneous mixture. Then the crucibles were kept inside the electric muffle furnace to heat the powder at 1100 ℃ for 1 h, the molten chemicals were stirred gently and carefully to get a bubble-free homogeneous mixture, and then cast on preheated (300℃) brass plate and pressed by another brass mold. The synthesised glasses have been transferred to a muffle furnace for annealing, by which thermal stresses can be removed. The obtained glasses are coded as BTNC0, BTNC1, BTNC2, BTNC3, BTNC4, and BTNC5 for y = 0, 0.1, 0.2, 0.3, 0.4, and 0.5 mol%, respectively. The composition of BTNC glasses, glass code, and images of glasses are tabulated in Table 1.

**Table 1:** Formulation of synthesised glass samples (mol%) and images of glass samples.

Sl. No	Glass Code	B_2_O_3_	TeO_2_	Na_2_O	CeO_2_	Image
1	BTNC0	65	25	10	0	
2	BTNC1	64.9	25	10	0.1	
3	BTNC2	64.8	25	10	0.2	
4	BTNC3	64.7	25	10	0.3	
5	BTNC4	64.6	25	10	0.4	
6	BTNC5	64.5	25	10	0.5	

### X-ray diffraction and FTIR characterization

The obtained glasses are converted into powder form with the help of an agate mortar and pestle. The powder samples are then used for XRD and FTIR characterization. The Bruker D8 Focus X-ray diffractometer has been used to probe the structure of the glasses. The XRD instrument was operated at room temperature, and Cu Kα radiations with a wavelength of 1.54 Å at 40 kV were used along with a scintillation detector, which measures the diffracted radiations (10°–80°) in the 2θ range. It is very important to find the functional groups existing in the obtained glass samples; therefore, powder samples are used in Fourier transform infrared spectroscopy. The Perkin Elmer FTIR Frontier 3 spectrophotometer was used to accomplish FTIR spectral analysis. It was scanned over the 400 cm^− 1^ – 4000 cm^–1^ spectral range at room temperature.

### Determination of physical properties

Density is one of the most important physical properties of a material; finding the density of a newly synthesised material is required for a material before using it for specific applications. The density of BTNC glasses is determined by Archimedes principle by using toluene as an immersion liquid; the density of toluene is 0.866 g/mL. In Eq. (1), ρ is the density of the sample, ρ_t_ is the density of toluene, *w*_*a*_ refers to the weight of the sample in air, and *w*_*t*_ is the weight of the sample in toluene.

1 $$\:\frac{\rho\:}{{\rho\:}_{t}}=\frac{{w}_{a}}{{w}_{a}-{w}_{t}}$$

Additionally, the other important physical properties, namely, molar volume, interionic distance, packing density, polaron radius, average boron-boron separation, oxygen packing density (OPD), RE ion concentration, field strength, and interionic concentration, were calculated by using appropriate formulae mentioned in references^38^.

The Eq. 

2 $$\:{V}_{m}=\frac{{M}_{w}}{\rho\:}$$

is used to estimate the molar volume ($\:{V}_{m}$) for every glass sample, where M_w_ represents the average molecular weight of the glass. The glass network is expected to alter with the incorporation of cerium oxide into the glass structure, and the mathematical equations given below were employed to investigate this change in physical properties.

3 $$\:<{d}_{B-B}>\:={\left(\frac{{V}_{m}}{2{N}_{A}\left(1-{X}_{B}\right)}\right)}^{1/3}$$

4 $$\:{N}_{i}=\frac{{N}_{A}\times\:mol\%\:of\:cation\times\:Valance\:of\:cation}{{V}_{m}}$$

5 $$\:OPD=\frac{1000\times\:O}{{V}_{m}}$$

6 $$\:{d}_{i}={\left(\frac{1}{{N}_{i}}\right)}^{\frac{1}{3}}$$

7 $$\:{R}_{p}=\frac{1}{2}{\left(\frac{\pi\:}{6{N}_{i}}\right)}^{\frac{1}{3}}$$

8 $$\:F=\frac{Z}{{\left({R}_{p}\right)}^{2}}$$

where the Avogadro number is represented by *N*_*A*_, and *X*_*b*_ represent percentage of B_2_O_3_ in each BTNC glass, *N*_*i*_ indicates the rare earth ion concentration, *O* signifies the number of oxygen atoms in the glass network, *d*_*i*_ represents the interionic distance, *R*_*p*_ is polaron radius, *F* stands for field strength, and *Z* is valency of the cation^39^.

### Determination of UV-vis spectroscopy and photoluminescence spectroscopy

The glass samples have been well-polished by emery papers of grades 500, 800, 1000, and 1500. The calculated thickness of the BTNC glass was 1.12 mm, and the calculated diameter of the glass was 1.05 cm, which were used for absorption edge studies through a Perkin Elmer Lambda-30 absorption spectrophotometer in the wavelength range of 200–1100 nm. Optical properties viz., direct energy band gap ($\:{E}_{g}^{d}$)_,_ indirect energy band gap ($\:{E}_{g}^{ind}$)_,_ molar refraction (R_m_), Urbach energy ($\:{E}_{u}$), steepness parameter (S), refractive index (n), dielectric constant (ε), reflection loss (R_L_), electronic molar polarizability (α_m_), and metallization criterion (M) of BTNC glasses were governed with appropriate mathematical equations^39^. Additionally, the optical basicity (Λ), electronic oxide polarizability $\:{(\alpha\:}_{O}^{2-})$, electronegativity (χ), and electric susceptibility (χ_e_), have been determined by means of literature reported by Dimitrov V, Sakka S, and Komatsu T^40,41^. The Horiba Jobin Yvon FluoroMax-4 spectrofluorometer was used to acquire steady-state fluorescence spectra at ambient temperature. The excitation source was a xenon arc lamp, and the emission detector was a photon-counting photomultiplier tube (PMT) model R928P. All spectra were recorded at the excitation wavelength of 486 nm.

### Estimation of refractive index, molar refraction, and metallization criterion

Refractive index is an important measurement and more practical for optical glasses. The refractive index of glasses was computed using the significant mathematical equations. In this equation, E_g_ represents the optical bandgap energy. Furthermore, the other optical properties mentioned in Sect. 3.4 are considered with knowledge of the refractive index^38,39,42^ and appropriate equations.

### Electronic polarizability, optical basicity, electronegativity, and electric susceptibility

The electronic oxide polarizability$\:\:{(\alpha\:}_{{O}^{2-}})\:$of BTNC glasses can be resolved by using an Eqs^42–44^. as given below.

9 $$\:{\alpha\:}_{{O}^{2-}}=\frac{\left[\left(\frac{{V}_{m}}{2.52}\right)\left(\frac{{n}^{2}-1}{{n}^{2}+2}\right)-\sum\:{\alpha\:}_{cat}\right]}{\left({N}_{{O}^{2-}}\right)}$$

where V_m_ represents the molar volume, n signifies the refractive index, $\:\sum\:{\alpha\:}_{cat}$ denotes the molar cation polarizability, and $\:{N}_{{\alpha\:}^{2-}}$ indicates the number of oxide ions. For any glass system having a general formula like uA_m_O_n_ + vB_o_O_p_ + wC_q_O_r_ + xD_s_O_t_, $\:\sum\:{\alpha\:}_{cat}$ can be calculated by u×m × α_A_ + v×o × α_B_ + w×q × α_C_ + x×s × α_D_ and $\:{N}_{{O}^{2-}}$ can be calculated by u×n + v×p + w×r + x×t. Henceforth, these obtained values are used to evaluate the electronegativity ($\:{\chi\:}_{e}$), optical basicity (Λ), and electric susceptibility (χ) with the equations as given below.

10 $$\:\varLambda\:=1.67\left(1-\frac{1}{{\alpha\:}_{{O}^{2-}}}\right)$$

11 $$\:\chi\:=\frac{\varLambda\:}{0.75}+0.25$$

12 $$\:{\chi\:}_{e}=\frac{\left({n}^{2}-1\right)}{4\pi\:}$$

## Results and discussion

### X-ray diffraction analysis

**Fig. 1:**
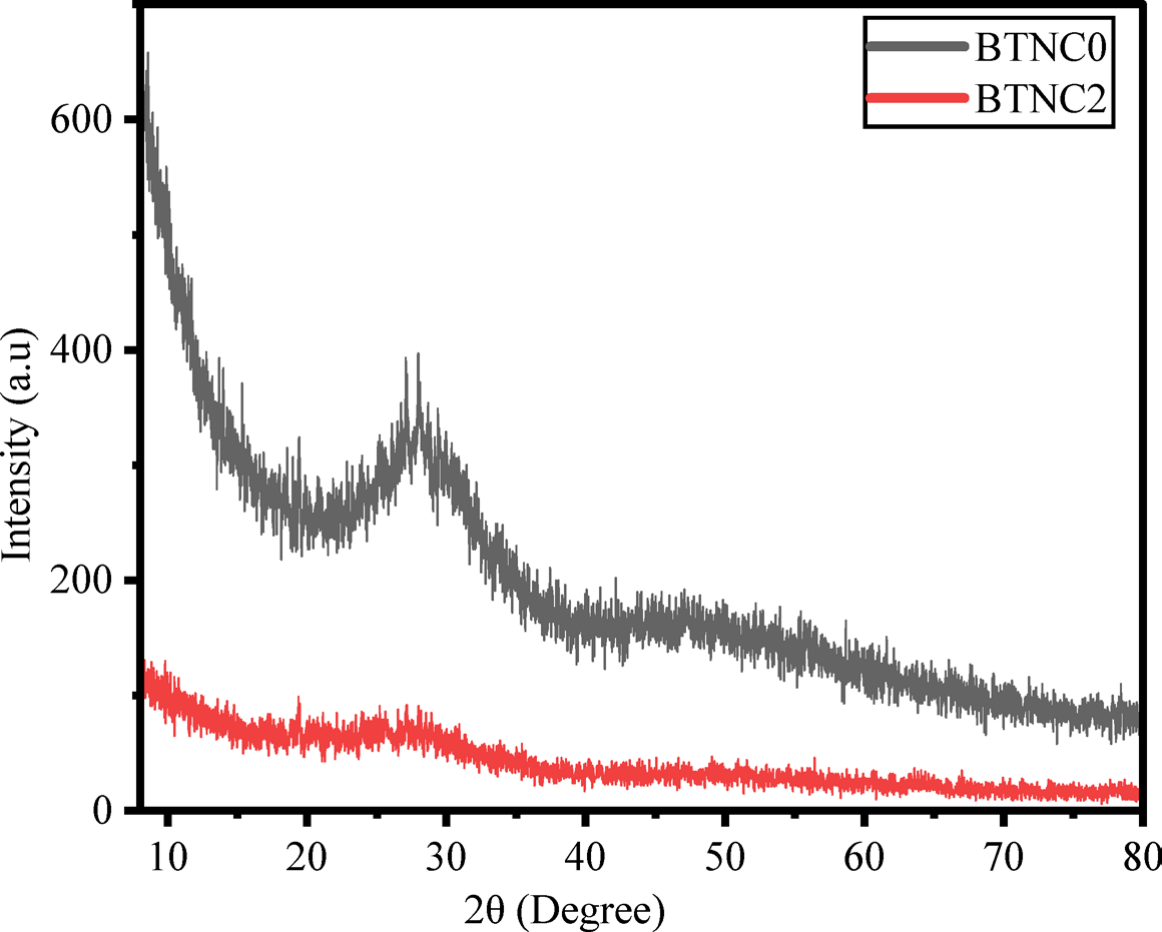
A typical XRD pattern of BTNC glasses.

The XRD patterns of the BTNC0 and BTNC2 (Undoped and CeO_2_-doped) glasses are depicted in Fig. 1. From Fig. 1, it is noticed that the absence of sharp crystalline peaks and the presence of broad and diffused peaks around 28° to 30°. In Fig. 1, the XRD pattern of BTNC0 exhibits a moderately broad diffraction peak, whereas BTNC2 shows a broad hump instead of a distinct peak. These variations can be ascribed to changes in the structural ordering and crystallinity of the samples. The presence of a broad peak in BTNC0 shows the presence of short-range crystalline order. In contrast, the broad hump noticed for BTNC2 suggests a highly disordered structure, where long-range periodicity is significantly reduced, and this arises due to compositional modifications with the addition of CeO_2_. Therefore, the change in the broad peak in BTNC0 to a broad hump in BTNC2 reflects the increase in the amorphous nature of the prepared glasses^45,46^.

### FTIR analysis

**Fig. 2:**
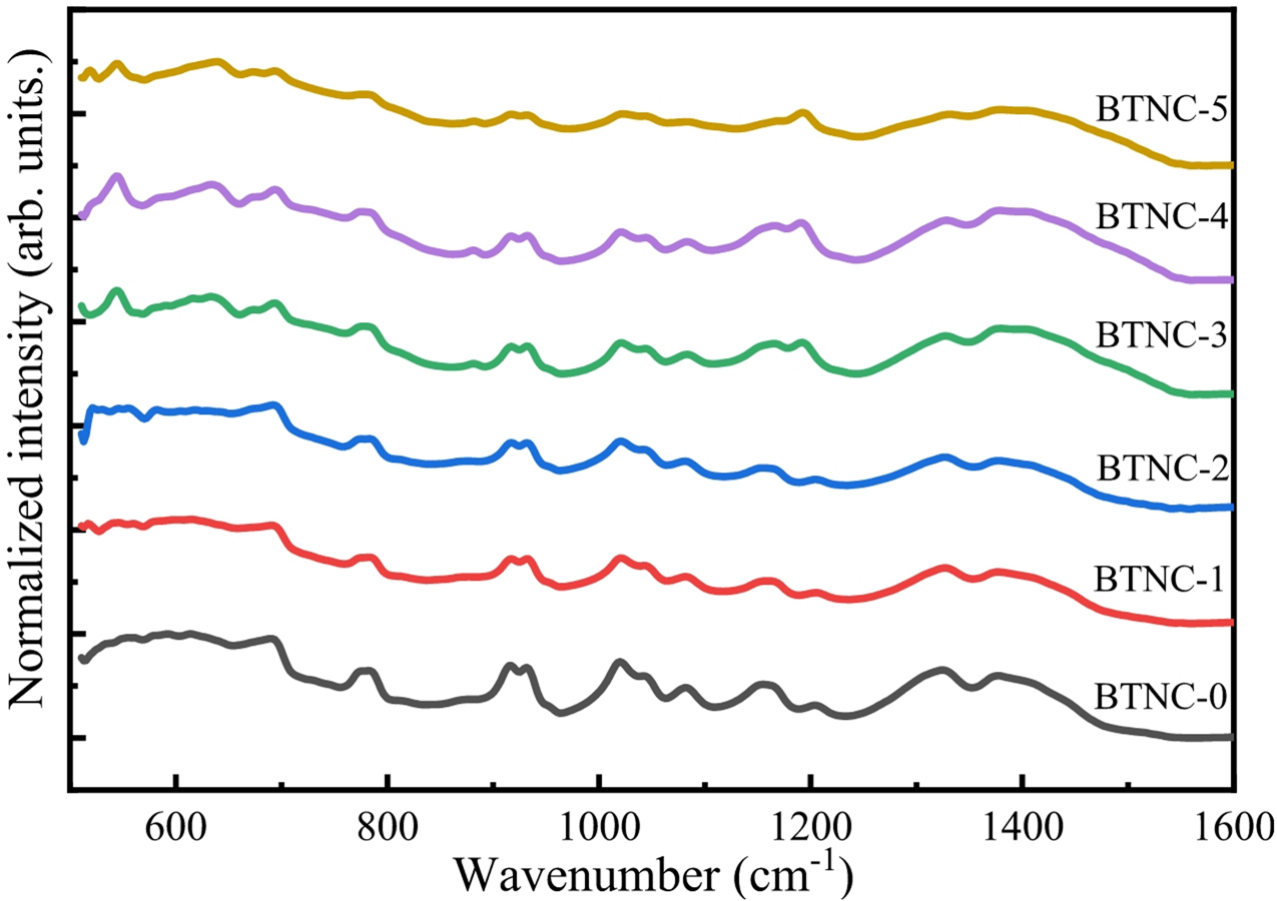
FTIR spectra of BTNC glasses.

The FTIR spectra for glass materials that are both cerium oxide-free and cerium oxide-doped are illustrated in Fig. 2. A prominent range for infrared absorption in glassy solids is seen between 1600 cm^− 1^ and 500 cm^− 1^. The entire band can be divided into four regions for a better understanding of the functional groups in the samples. The first region is taken between 500 cm^− 1^ and 600 cm^− 1^ vibration modes of sodium cations. The second region is identified between 600 cm^− 1^ and 800 cm^− 1^, which can be assigned to O_3_B-O-BO_3_ bonds bending vibrations. In this region, the occurrence of bending vibrations of B-O-B in the borate network is associated with the presence of Te-O-B and Ce-O-B cross-linkages, including stretching vibrations of the Te-O in TeO_4_ and TeO_3_ units. The third region is seen between 800 cm^− 1^ and 1150 cm^− 1^, which is attributed to the presence of B-O stretching present in BO_4_ units. The fourth region chosen is between 1150 cm^− 1^ and 1600 cm^− 1^, which indicates the existence of BO_3_ units in the asymmetric stretching vibration mode^47–49^.

**Fig. 3:**
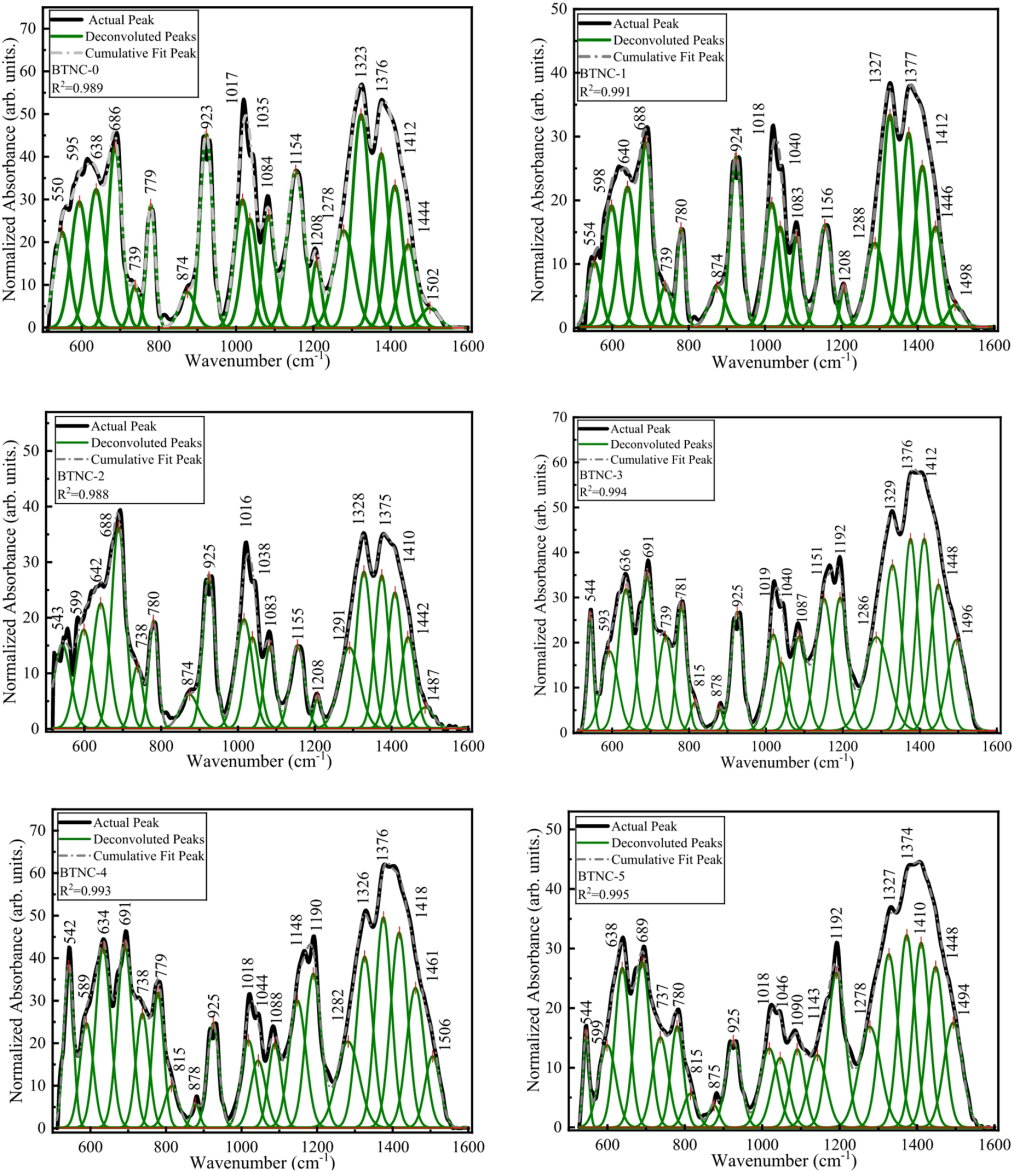
Deconvoluted spectra of BTNC glasses.

To determine the different structural units of borate and tellurite, the FTIR spectra were deconvolved employing the Multiple Peak Fit features in Origin 2025 software and a correlation coefficient (R²) of 0.99. The deconvolution technique allows the breakdown of overlapped spectral bands into individual ones. The broad and asymmetric absorption bands in the given spectral range are explained with respect to Fig. 3, and the related FTIR peak assignments are compiled in Table 2. The peak at 542 cm^− 1^ – 554 cm^− 1^ is ascribed to vibrations of sodium cations^50,51^. The peaks at 589 cm^− 1^ – 599 cm^− 1^ are attributed to the vibration of the continuous network, which consists of TeO_4_ trigonal bipyramids (tbp)^52,53^. The peaks 634 cm^− 1^ to 642 cm^− 1^ are ascribed to the vibration of the cross-linkages between CeO_4_ and BO_3_, Ce-O-B and Te-O-B bonds in TeO_4_ units^54–56^. The peak 686 cm^− 1^ to 691 cm^− 1^ is attributed to stretching vibrations of TeO_3_/TeO_3+1_ units of tellurium with bridging oxygen (BO) of B – O – B bending vibrations^53^. The peak at 737 cm^− 1^ to 739 cm^− 1^ is ascribed to stretching modes of the TeO_3_ and TeO_3+1_ forming non-bridging oxygens (NBOs)^53^. The peaks at 781 cm^− 1^ to 779 cm^− 1^ and 815 cm^− 1^ are attributed to the (Te_eq_–O)_s_ and (Te_eq_–O) as vibrational modes of TeO_3_ trigonal pyramid (tp) or TeO_3+1_ polyhedra units^53,57^. The peak 874 cm^− 1^ to 878 cm^− 1^ is ascribed to B-O-B bending of tri-, tetra-, and penta-borate units^42,58^. The peaks at 923 cm^− 1^ to 925 cm^− 1^ and 1016 cm^− 1^ to 1019 cm^− 1^ are associated with diborate units [B_4_O_7_^2−^] of BO_4_ or [BØ_4_]^−^ groups^42,55^. The peaks at 1035 cm^− 1^ to 1044 cm^− 1^, 1083 cm^− 1^ to 1090 cm^− 1^, and 1143 cm^− 1^ to 1156 cm^− 1^ are ascribed to stretching vibrations and overlapping vibrations of B-O units due to tri-, tetra-, and penta-borate groups^48^. The peaks at 1190 cm^− 1^ – 1208 cm^− 1^ are associated with the asymmetric stretching vibration of BO_3_^3−^ (ortho-borate) units^48^. The peaks at 1278 cm^− 1^ to 1291 cm^− 1^ and 1323 cm^− 1^ to 1329 cm^− 1^ are attributed to the asymmetric stretching vibration of B_2_O_5_^4−^ (pyro-borate unit)^48^. The peaks at 1374 cm^− 1^ to 1376 cm^− 1^ are attributed to asymmetric stretching vibration of B-O^−^ in BØ_2_O^−^ (meta-borate) units^48^. The peaks 1410 cm^− 1^ to 1418 cm^− 1^ and 1442 cm^− 1^ to 1461 cm^− 1^ are ascribed to stretching vibrations of B-Ø in BØ_3_ units^50^. The peaks from 1487 cm^− 1^ to 1506 cm^− 1^ are attributed to asymmetric stretching relaxation of trigonal BO_3_ units (B-O bonds) with NBOs^49,50^. An increase in NBOs was observed as the CeO_2_ concentration increased in the glass system.

**Table 2:** FTIR peak assignments of BTNC glasses.

BTNC-0	BTNC-1	BTNC-2	BTNC-3	BTNC-4	BTNC-5	Band assignments	Refs.
550	554	543	544	542	544	Vibrations of sodium cations.	^50,51^
595	598	599	593	589	599	Vibration of the continuous network, which consists of TeO_4_ (tbp).	^52,53^
638	640	642	636	634	638	Vibration of the cross-linkages between CeO_4_ and BO_3_, Ce-O-B, and Te-O-B bonds in TeO_4_ units.	^54^
686	688	688	691	691	689	Stretching vibrations of tellurium with BO of TeO_3_/TeO_3+1_ units with B–O–B bending vibrations.	^53,55^
739	739	738	739	738	737	Stretching modes of NBOs found in the TeO_3_ and TeO_3+1_ units.	^53^
779	780	780	781	779	780	the (Te_eq_–O)_s_ and (Te_eq_–O) as vibrational modes of TeO_3_ trigonal pyramid (tp) units or TeO_3+1_ polyhedra.	^53,57^
-	-	-	815	815	815		
874	874	874	878	878	875	B-O-B bending of tri-, tetra-, and penta-borate units.	^42,58^
923	924	925	925	925	925	Diborate units [B_4_O_7_^2−^] of BO_4_ or [BØ_4_]- groups.	^42,55^
1017	1018	1016	1019	1018	1018		
1035	1040	1038	1040	1044	1046	Stretching vibrations and overlapping vibrations of B-O units due to tri-, tetra- and penta- borate groups.	^48,55,56^
1084	1083	1083	1087	1088	1090		
1154	1156	1155	1151	1148	1143		
1208	1208	1208	1192	1190	1192	Asymmetric stretching vibration of BO_3_^3−^ (ortho-borate) units.	^48,55^
1278	1288	1291	1286	1282	1278	Asymmetric stretching vibration of B_2_O_5_^4−^ (pyro-borate unit).	^48,55^
1323	1327	1328	1329	1326	1327		
1376	1377	1375	1376	1376	1374	Asymmetric stretching vibration of B-O^−^ in BØ_2_O^−^ (meta-borate) units.	^48,55^
1412	1412	1410	1412	1418	1410	Stretching vibrations of B-Ø in BØ_3_ units.	^50^
1444	1446	1442	1448	1461	1448		
1502`	1498	1487	1496	1506	1494	Asymmetric stretching relaxation of trigonal BO_3_ units (B-O bonds) with NBOs.	^49,50^

### Physical properties

#### Density ($\:\boldsymbol{\rho\:}$), Molar volume ($\:{\boldsymbol{V}}_{\boldsymbol{m}}$), RE ion concentration (N_i_), average boron-boron separation (< d_B-B_>), and oxygen packing density (OPD)

**Fig. 4:**
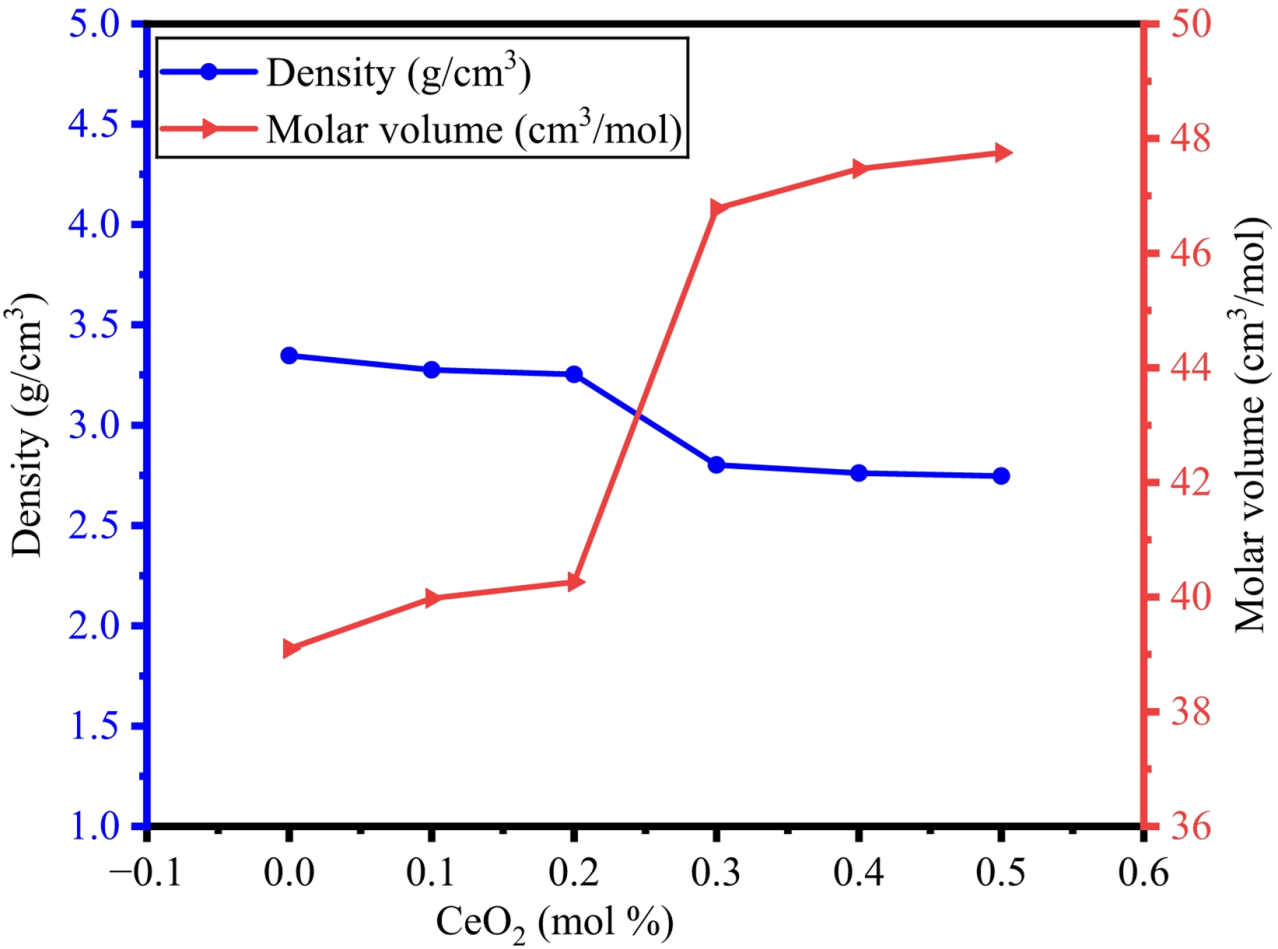
Variation of density and molar volume of BTNC glasses.

One of the most important and basic things to understand how the glass properties vary is investigating their internal structure. Determination of the density of the synthesised glass material is very much required to figure out structural modification and compactness of the materials. The density of the BTNC glasses has been measured by using Eq. (1) and toluene as an immersion liquid. Similarly, the molar volume of the glasses was calculated by using Eq. (2). As anticipated and presented in the literature, the density and molar volume show reverse behaviour, as shown in Table 3. It was noticed from Fig. 4 that the insertion of CeO_2_ into the glass matrix altered the density and compactness of the glass. The density of the glass mostly depends on the molecular weight, the quantity of each oxide in the glass system, and the compactness of the structure of the glass^36^. As can be realised from Fig. 4, adding cerium oxide to the origins of the BTNC glass samples, density decreases from 3.347 g/cm^− 3^ to 2.746 g/cm^3^ as shown in Table 3. The significant change in density was attributed to (i) the variation of molar mass of cerium oxide^59,60^, (ii) greater changes in the average molecular weight of CeO_2_ than B_2_O_3_, and (iii) the Presence of non-variable Na_2_O_3_ (Best glass network modifier) in the glass matrix^50,51^. The decrease in density as the CeO_2_ concentration increased from 0.1 mol % to 0.5 mol % indicates that the BTNC glass, which contains more NBOs, exhibits a weaker network; in some of the glass networks, the same kind of results have been reported^59^. Due to the production of NBOs, the increase in molar volume is observed in BTNC glasses^60^.

**Fig. 5:**
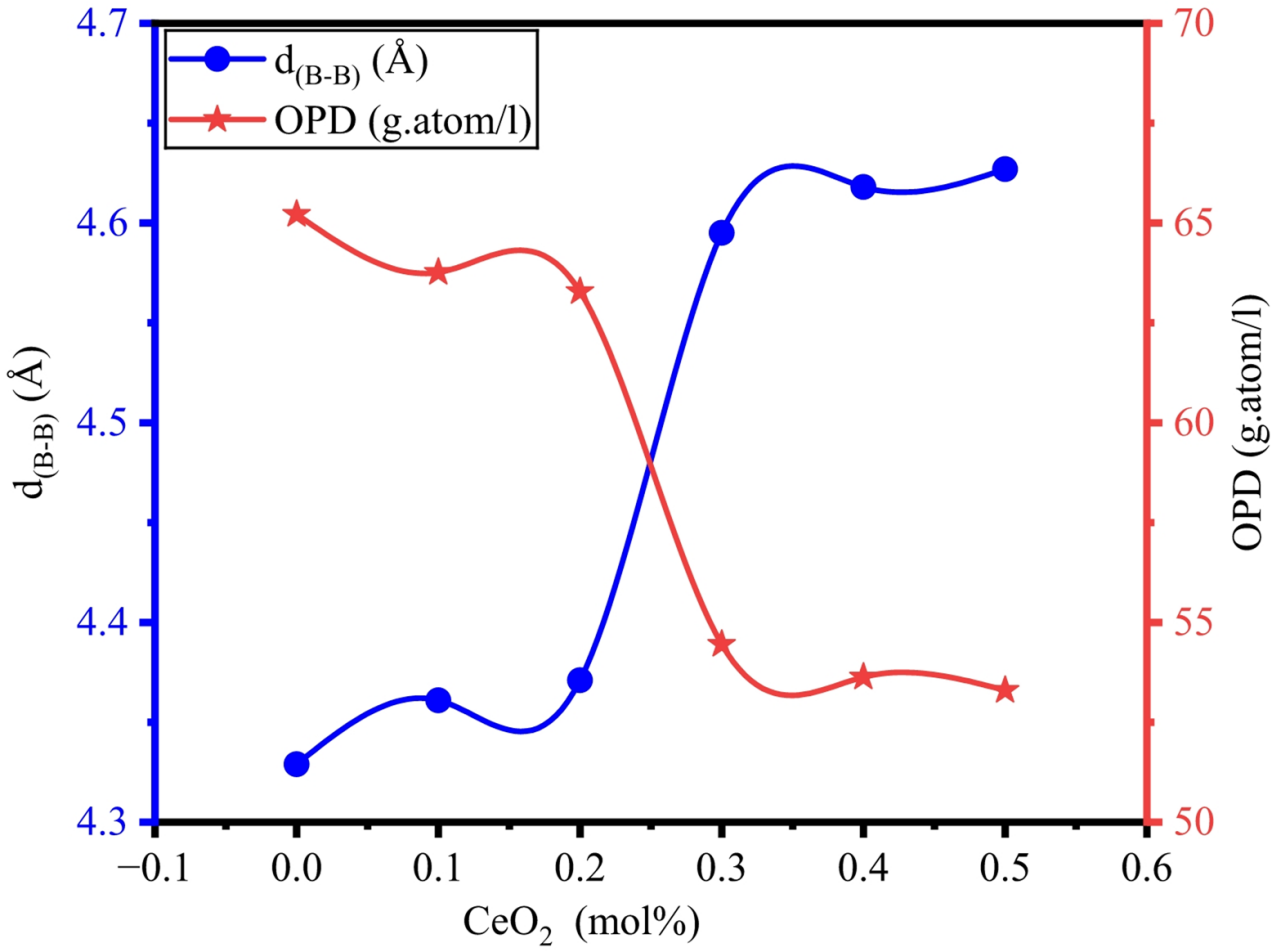
Variation of d_(B−B)_ and OPD of BTNC glasses.

The impact of CeO_2_ content on the glass network can be examined with the knowledge of the average boron-boron separation (< d_B−B_>). The outcome values of < d_B−B_> vary with the content of CeO_2_, as shown in Fig. 5. The measured values of < d_B−B_> range from 4.329 Å to 4.628 Å, as depicted in Table 3. The existence of cerium oxide leads to an increase in the average (< d_B−B_>) separation, subsequently contributing to a change in the molar volume^61,62^. In every oxide or metal oxide glass, the array of atoms of oxygen can be extracted by the oxygen packing density (OPD). The improvement in CeO_2_ doping aids the modifications in the molar volume, and it also denotes the hardness of the oxide glasses. The NBOs and the glass structure varied with the increase in CeO_2_ content. The leeway of the glass structure is in good agreement with the rise of CeO_2_^57,61^.

#### Polaron radius ($\:{\boldsymbol{R}}_{\boldsymbol{p}}$), interionic distance ($\:{\boldsymbol{d}}_{\boldsymbol{i}}$), field strength ($\:\boldsymbol{F}$), and packing density ($\:{\boldsymbol{V}}_{\boldsymbol{d}}$)

**Fig. 6:**
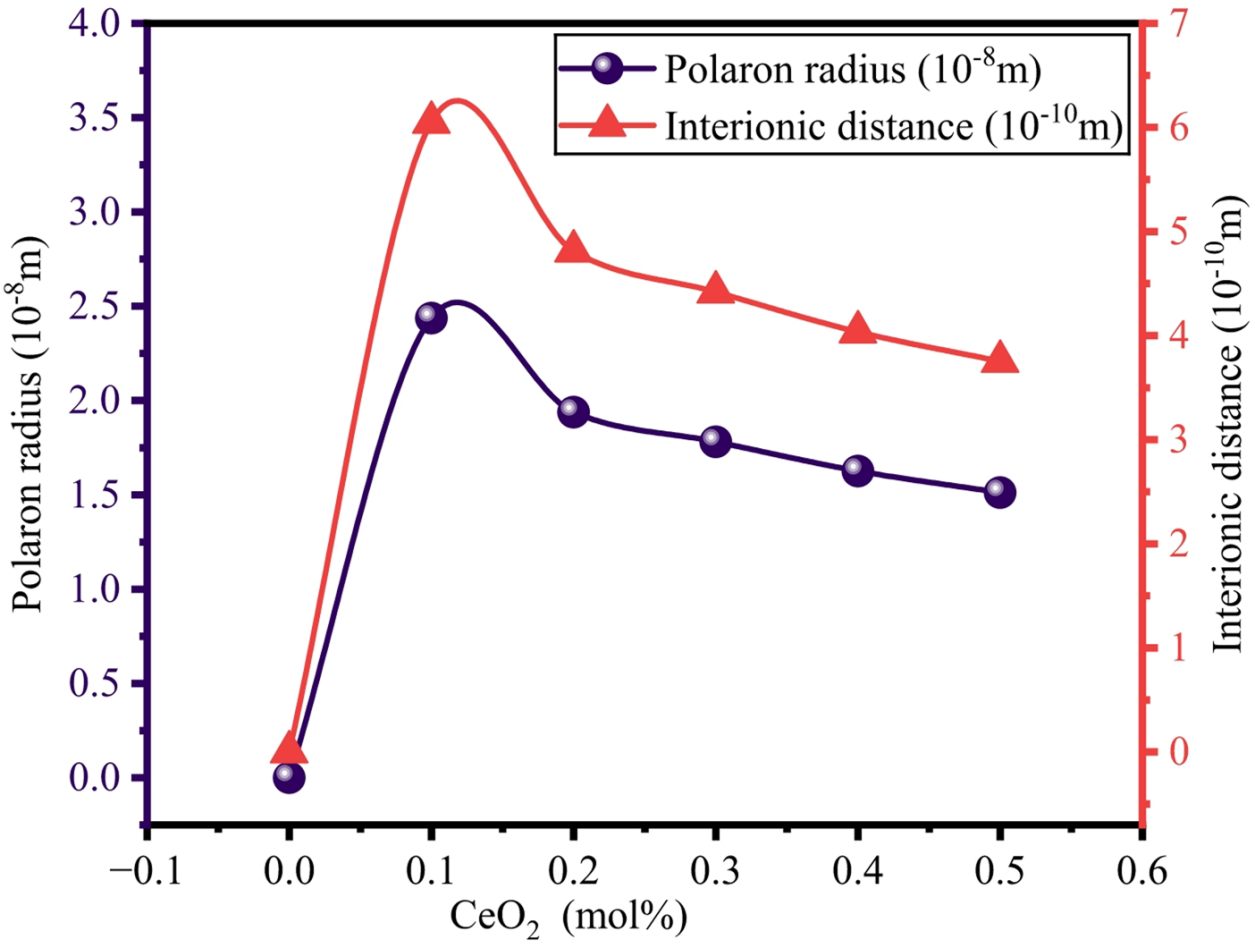
Variation of polaron radius and interionic distance of BTNC glasses.

**Fig. 7:**
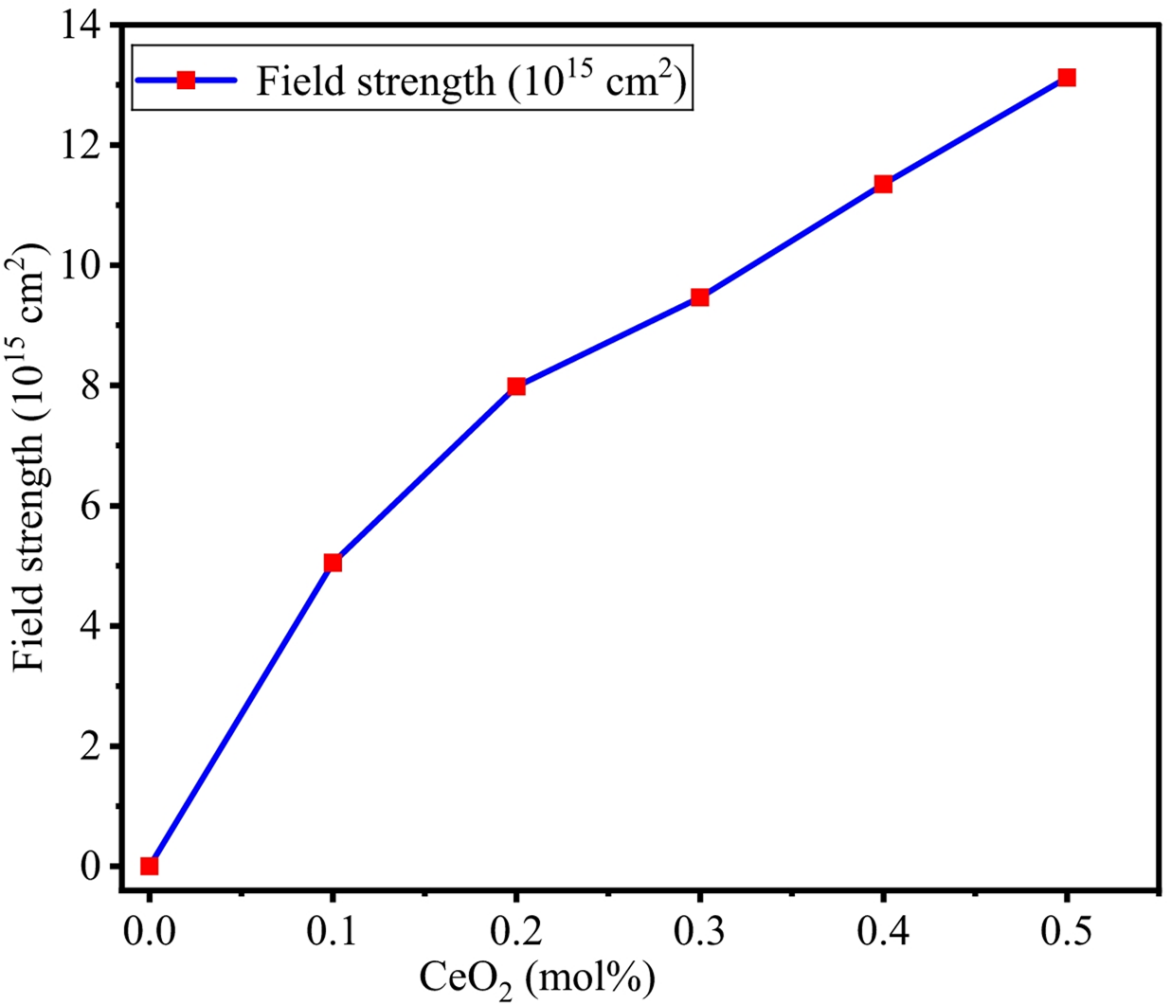
Variation of the field strength of BTNC glasses.

Important physical parameters, such as polaron radius, interionic distance, field strength, and packing density, are calculated by using mathematical Eqs. 6, 7, 8, and 13, respectively. The obtained values are tabulated in Table 3. Figure 6 depicts the relation between the polaron radius and interionic distance versus the concentration of CeO_2_. It is perceived from Fig. 6 that the polaron radius varies with increasing CeO_2_, which is due to diminished and distorted lattice sites and decreasing interionic separations^62^. Field strength of the BTNC glasses measured by Eq. (8) and values are tabulated in Table 3 and shown in Fig. 7. From Fig. 7, it was observed that the field strength is increased with increasing CeO_2_ content, owing to an increase in the attractive forces between ions and with neighbouring structural units of CeO_2_^55,57^.

The facts of the rigidity of the oxide glass system can be found by the packing density ($\:{V}_{d}$), the mathematical relation to be determined is taken from the literature^63^,

13 $$\:{V}_{d}=\frac{1}{{V}_{m}}\sum\:{V}_{i}{X}_{i}$$

Where V_i_ is the packing factor and X_i_ mole fraction of the matrix.

The $\:{V}_{i}$ can be calculated by using ionic radii of some oxide A_x_O_y_, having A and O ions with ionic radii r_A_ and r_O,_ by using the Eq. (14):

14 $$\:{V}_{i}=\frac{4\pi\:{N}_{A}}{3}\left({Xr}_{M}^{3}+{Yr}_{O}^{3}\right)$$

**Fig. 8:**
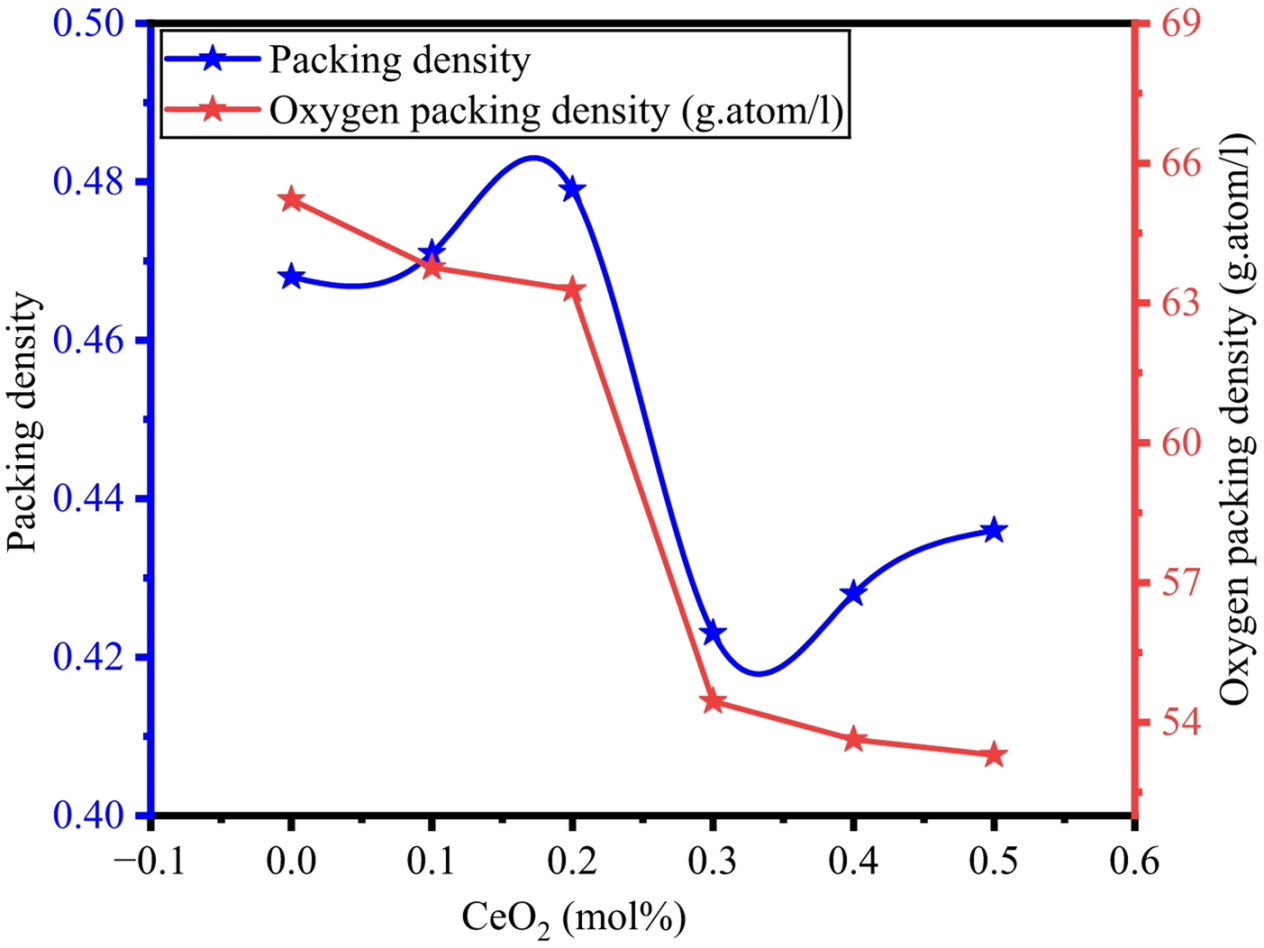
Variation of packing density and oxygen packing density of BTNC glasses.

V_i_ of B_2_O_3_. Na_2_O_3_, TeO_2_, and CeO_2_ are derived from the literature^63^ i.e., 20.8, 11.2, 14.7, and 52.29 cm^3^/mol, respectively. Figure 8 illustrates the variations of Packing density $\:{V}_{d}$ and OPD versus CeO_2_ mol%. It is observed from Fig. 8 that the $\:{V}_{d}$ varying significantly with rising CeO_2_ content, which causes the increase in the molar volume values, indicating variations in the rigidity of the glasses.

Furthermore, the number of bonds per unit volume of the BTNC glasses was found by using a mathematical relation^63^:

15 $$\:{n}_{b}=\frac{{N}_{A}}{{V}_{m}}m$$

Where m denotes the average coordination number of the BTNC glass, and it is given by

16 $$\:m={\sum\:}_{i}C{N}_{i}{X}_{i}$$

Here, CN_i_ refers to the coordination number of the cations that are directly taken from the literature^63^. From the obtained values, it is noted that the number of bonds per unit volume of glass is decreasing with increasing quantity of CeO_2_, which agrees with the trend observed in the density and molar volume values.

**Table 3:** Physical properties of BTNC glasses with error ± 0.001.

Glass code	$\:{\boldsymbol{M}}_{\boldsymbol{w}}$, (g/mol)	$\:\boldsymbol{\rho\:}$, (g/cm^3^)	$\:{\boldsymbol{V}}_{\boldsymbol{m}}$, (cm^3^/mol)	$\:{\boldsymbol{N}}_{\boldsymbol{i}}$ × 10^21^, (ions/cm^3^)	$\:{\boldsymbol{d}}_{\boldsymbol{i}}$, (nm)	$\:<{\boldsymbol{d}}_{\boldsymbol{B}-\boldsymbol{B}}>$, (Å)	$\:\boldsymbol{O}\boldsymbol{P}\boldsymbol{D}$, (g.atom/l)	$\:{\boldsymbol{R}}_{\boldsymbol{p}}$, (Å)	$\:\boldsymbol{F}$ × 10^15^, (cm^− 2^)	$\:{\boldsymbol{n}}_{\boldsymbol{b}}$ × 10^28^, (m^− 3^)	$\:{\boldsymbol{V}}_{\boldsymbol{d}}$
BTNC0	130.869	3.347	39.100	0	0	4.329	65.217	0	0	6.161	0.468
BTNC1	130.926	3.275	39.977	4.520	0.605	4.362	63.761	2.437	5.051	6.110	0.471
BTNC2	130.974	3.253	40.263	8.976	0.481	4.372	63.285	1.939	7.981	6.150	0.480
BTNC3	131.023	2.801	46.777	11.588	0.442	4.596	54.450	1.781	9.463	5.366	0.424
BTNC4	131.072	2.761	47.473	15.225	0.403	4.619	53.631	1.626	11.351	5.358	0.428
BTNC5	131.121	2.746	47.750	18.92	0.375	4.628	53.299	1.512	13.121	5.398	0.436

### Optical properties

#### Direct band gap ($\:{\boldsymbol{E}}_{\boldsymbol{g}}^{\boldsymbol{d}}$), Indirect band gap ($\:{\boldsymbol{E}}_{\boldsymbol{g}}^{\boldsymbol{i}\boldsymbol{n}\boldsymbol{d}}$), Urbach energy ($\:{\boldsymbol{E}}_{\boldsymbol{u}}$), and Steepness parameter (S)

The absorption coefficient α(ν) was measured at the photon energies near the absorption edge for all the glass samples. According to the reported literature, an Eq. (17) put forth by Davis and Mott corresponds to both direct and indirect band gap data.

17 $$\:\alpha\:\left(h\vartheta\:\right)=\left(\frac{B}{h\vartheta\:}\right){\left(h\vartheta\:-{E}_{opt}\right)}^{m}$$

18 $$\:\alpha\:\left(\lambda\:\right)=2.303\left[\frac{A}{d}\right]$$

**Fig. 9:**
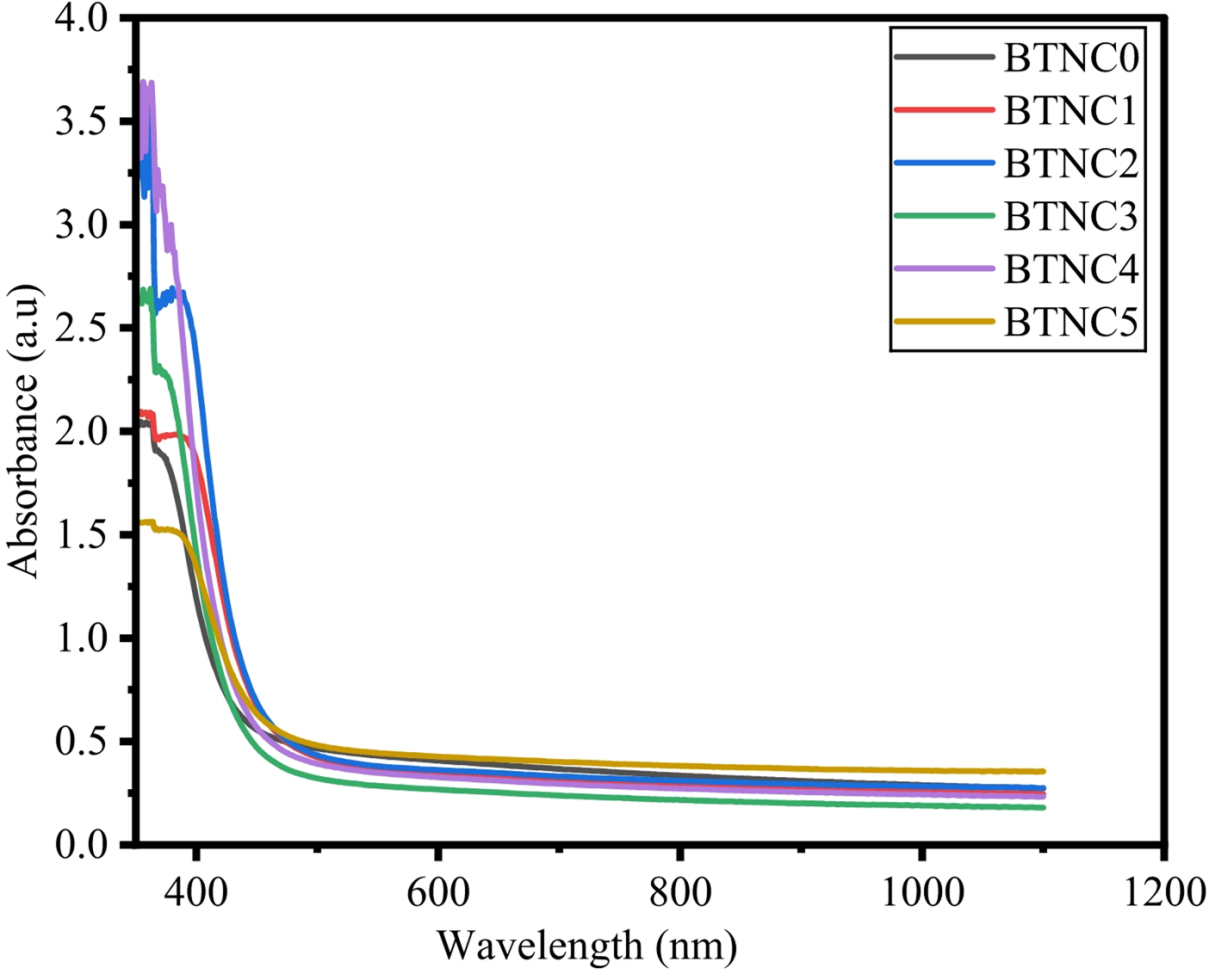
UV-absorption spectra of BTNC glasses.

**Fig. 10:**
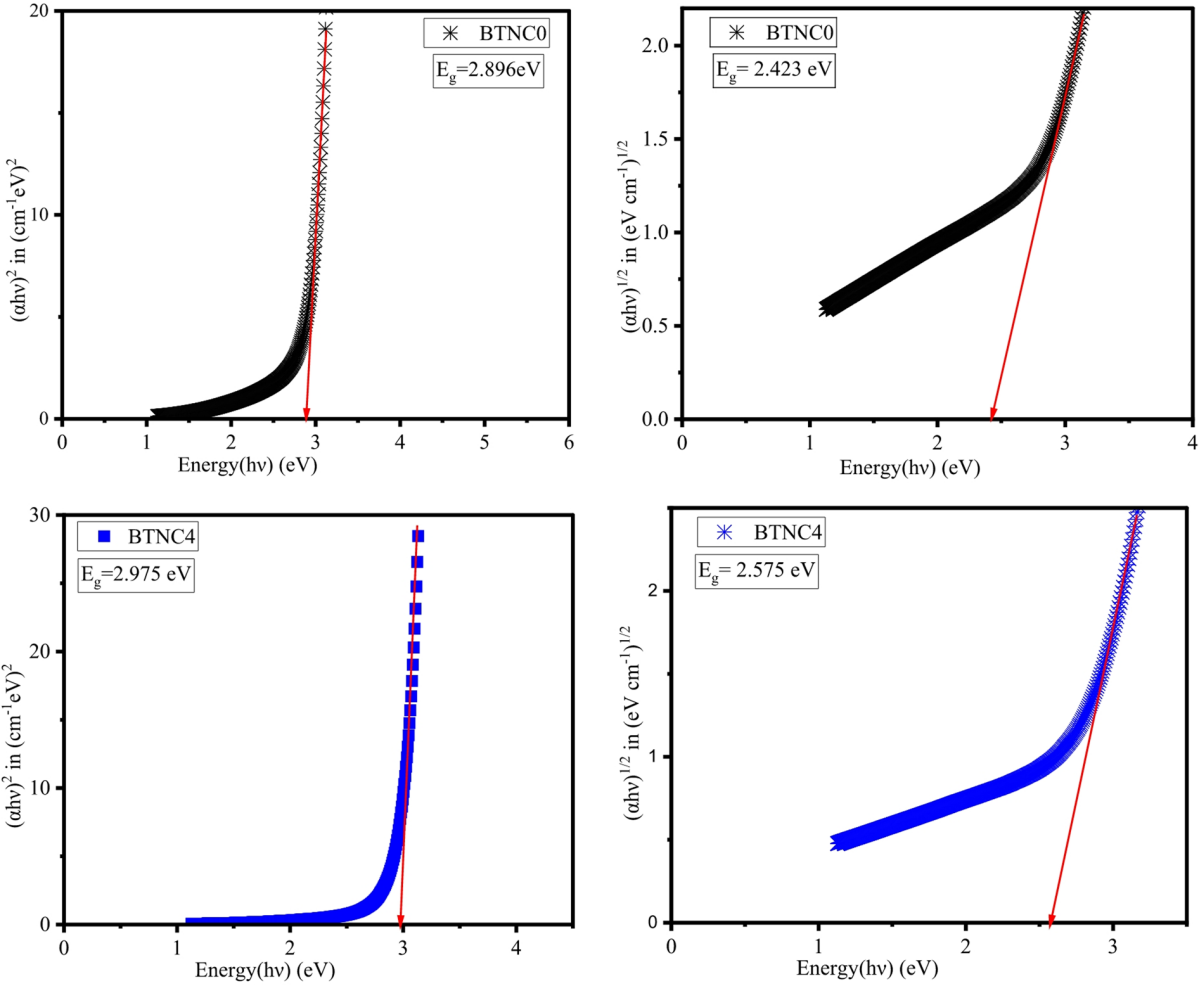
Typical graphs of the direct and indirect band gap of BTNC glasses.

**Fig. 11:**
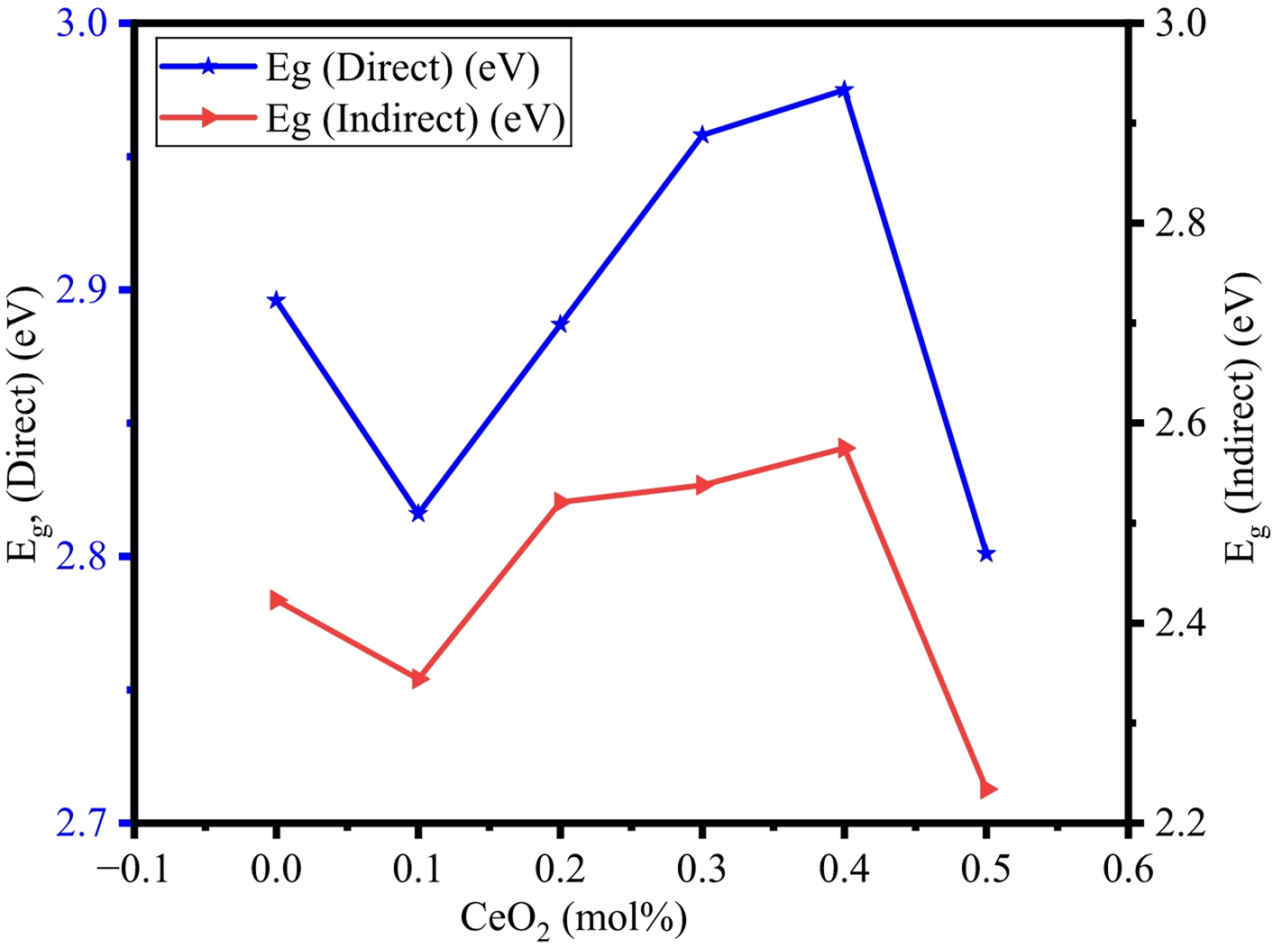
Variation of direct and indirect energy band gaps of BTNC glasses.

Where A denotes absorbance, d refers to the thickness of BTNC glass samples, and m is defined as the index, which can be selected by the following values such as 1/2, 3/2, 2, and 3, corresponding to direct allowed, direct forbidden, indirect allowed, and indirect forbidden, respectively^43,56,64^. $\:h\vartheta\:$ indicates photon energy, and E_opt_ refers to optical band gap energy. Figure 9 shows the UV-absorption spectra of BTNC glasses. Figure 10 represents the typical Taucs plots for the estimation of the direct and indirect band gap values. The direct and indirect energy band gap values are listed in Table 4. The attained direct band gap values are in the range of 2.801 eV to 2.975 eV, and the indirect band gap values range between 2.234 eV to 2.575 eV. The lowest E_g_ value, 2.801 eV, is recorded for BTNC5 glass, and the highest value, 2.975 eV, is recorded for BTNC4 glass. The outcome of the energy band gap values indicates the impact of CeO_2_, which is due to a modification in the structure due to an increase in the formation of NBOs. In addition, as observed from the FTIR, incorporation of CeO_2_ leads towards the formation of (tp) units or TeO_3+1_ polyhedra and meta-borate in BØ_3_ units leads to the formation of NBOs in the BTNC glass network. The correlation between the direct band gap and the indirect band is shown in Fig. 11. The quantity of structural disorder in the glass structure can be found by finding one significant parameter, that is, Urbach energy (E_u_). The investigation of E_u_ of the density of states is the degree of the width of the delocalized electrons.

The exponential increase in α(λ) is the consequence of the absorption of photons possessing higher energy than the band gap energy. The Urbach energy of the prepared glasses was calculated by Eq. (19) and by the inverse of the obtained slope value by plotting a graph between ln(α) and hϑ^64,65^.

19 $$\:ln\alpha\:=ln{\alpha\:}_{o}+\frac{h\vartheta\:}{{E}_{u}}$$

**Fig. 12:**
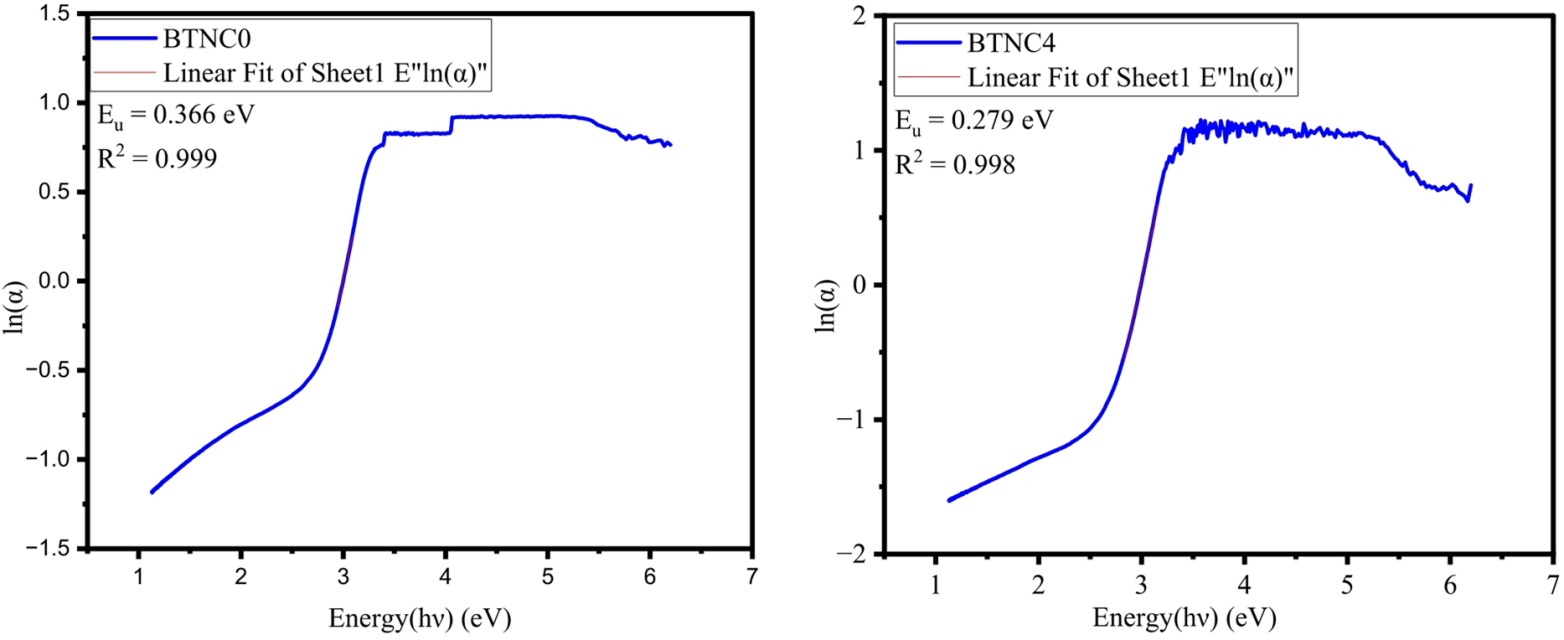
Typical graphs of urbach energy of BTNC glasses.

**Fig. 13:**
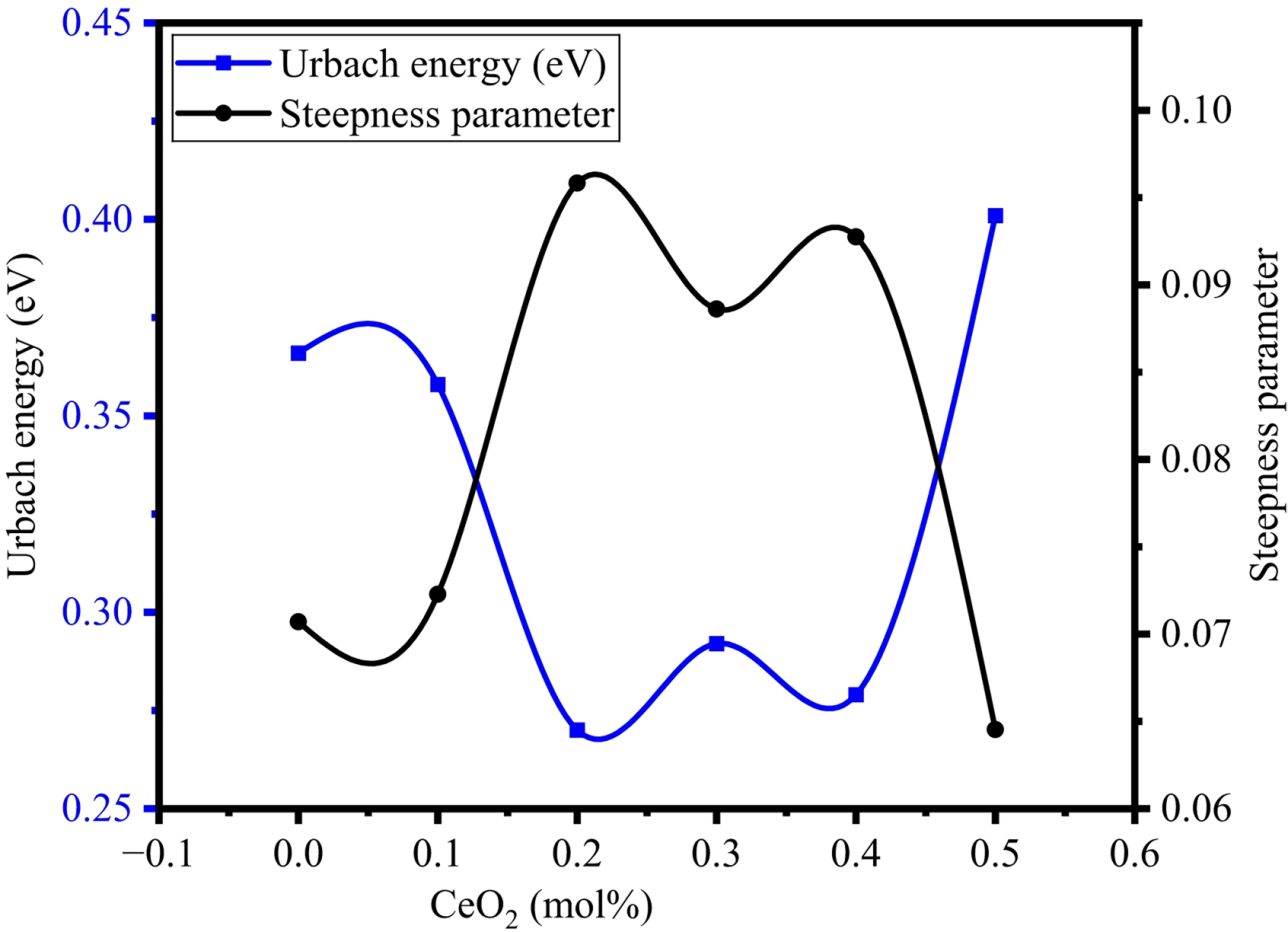
Variation of Urbach energy and steepness parameter of BTNC glasses.

Table 4 contains the obtained values of E_u_, where an increase in the CeO_2_ quantity has inclined the Urbach energy values to undergo variation between 0.270 and 0.401. The higher E_u_ values indicate more structural disorder in the glass structure, which is also related to the less compactness and more NBOs in the BTNC glass system. Figure 12 represents the typical graphs to determine the Urbach energy of BTNC glasses. Figure 13 shows the variation of Urbach energy and steepness parameter with CeO_2_. The steepness parameter, which gives the broadening of the optical absorption edge produced with excitation-phonon or electron-phonon interactions^64,65^, has been calculated by using the Urbach energy:

20 $$\:S=\frac{{K}_{B}T}{{E}_{u}}$$

Where K_B_ and T refer to the Boltzmann constant and room temperature, respectively. Table 4 shows the obtained values of $\:S$, where an increase in the CeO_2_ quantity has varied the $\:S$ values between 0.064 and 0.095.

**Table 4:** Direct band gap ($\:{E}_{g}^{d}$), Indirect band gap ($\:{E}_{g}^{ind}$), Urbach energy ($\:{E}_{u}$), and Steepness parameter ($\:S$) with error $\:\pm\:$0.001.

Glass Code	$\:{\boldsymbol{E}}_{\boldsymbol{g}}^{\boldsymbol{d}}\:$(eV)	$\:{\boldsymbol{E}}_{\boldsymbol{g}}^{\boldsymbol{i}\boldsymbol{n}\boldsymbol{d}}$ (eV)	$\:{\boldsymbol{E}}_{\boldsymbol{u}}$ (eV)	S
BTNC0	2.896	2.423	0.366	0.070
BTNC1	2.816	2.344	0.358	0.072
BTNC2	2.887	2.521	0.270	0.095
BTNC3	2.958	2.538	0.292	0.088
BTNC4	2.975	2.575	0.279	0.092
BTNC5	2.801	2.234	0.401	0.064

#### Refractive index, molar refraction, molar electronic polarizability and Numerical aperture

The evaluation of optical properties through its refractive index parameters is essential for selecting any glass for optical fibre applications and other optical material applications. It is well known that light travels in straight lines until it strikes a polished surface which divides two transparent media. Dimitrov and Sakka^40^ proposed a method to estimate the refractive index of BTNC glass systems with the help of optical band gap energies, i.e., Eq. (21).

21 $$\:\frac{{n}^{2}-1}{{n}^{2}-2}=1-\sqrt{\frac{{E}_{g}}{20}}$$

Additionally, there are a few other methods also available to find the RI of the glasses, as given below, and the obtained values are compared with each other.

Kumar-Singh proposed^66^ a relation for finding RI by using the energy band gap values of glasses:

22 $$\:{n}_{K-S}=3.3669{E}_{g}^{-0.32234}$$

Herve and Vandamme proposed another relation for RI^67^.

23 $$\:{n}_{H-V}=\sqrt{1+{\left(\frac{13.6}{{E}_{g}+3.47}\right)}^{2}}$$

and Reddy and Sakka relation (Nr-s)^68^.

24 $$\:{n}_{R-S}=-0.73\mathrm{ln}\left(0.0274176{E}_{g}+0.5511\right)$$

The RI of BTNC glasses and tabulated in Table 5. It is noticed that the RI of the glasses were closely related, and the average value of this is taken. The RI of the glasses has undergone significant variations along with the rise of CeO_2_ content. This is due to the formation of the NBOs and a considerable change in the density of the BTNC glass system. The refractive index values of BTNC glasses are relatively higher than those of other glasses, like boro-bismuth tellurite glasses^48^, borate glasses^4^, and alkali borate glasses^50^.

The molar refraction (R_m_) of the BTNC glasses was calculated by the mathematical Eq. (25), known as the Lorentz-Lorentz equation. The molar refraction is the parameter that gives the average molar refraction of the isotropic materials.

25 $$\:{R}_{m}=\frac{{n}^{2}-1}{{n}^{2}+2}{V}_{m}$$

26 $$\:{\alpha\:}_{m}=\frac{{R}_{m}}{2.52}\:\:$$

The molar electronic polarizability (α_m_) is the parameter associated with molar refraction as given in Eq. (26). The obtained R_m_ and α_m_ values are depicted in Table 5. It is noted that both R_m_ and α_m_ values are considerably increased with the addition of CeO_2_ quantity. This can be attributed to the formation of NBOs; hence, the BTNC glasses are significantly polarised in the glass network^69^.

The numerical aperture (NA) of the BTNC glasses was evaluated by a mathematical relation:

27 $$\:NA=n{\left[2\varDelta\:\right]}^{\frac{1}{2}}$$

**Fig. 14:**
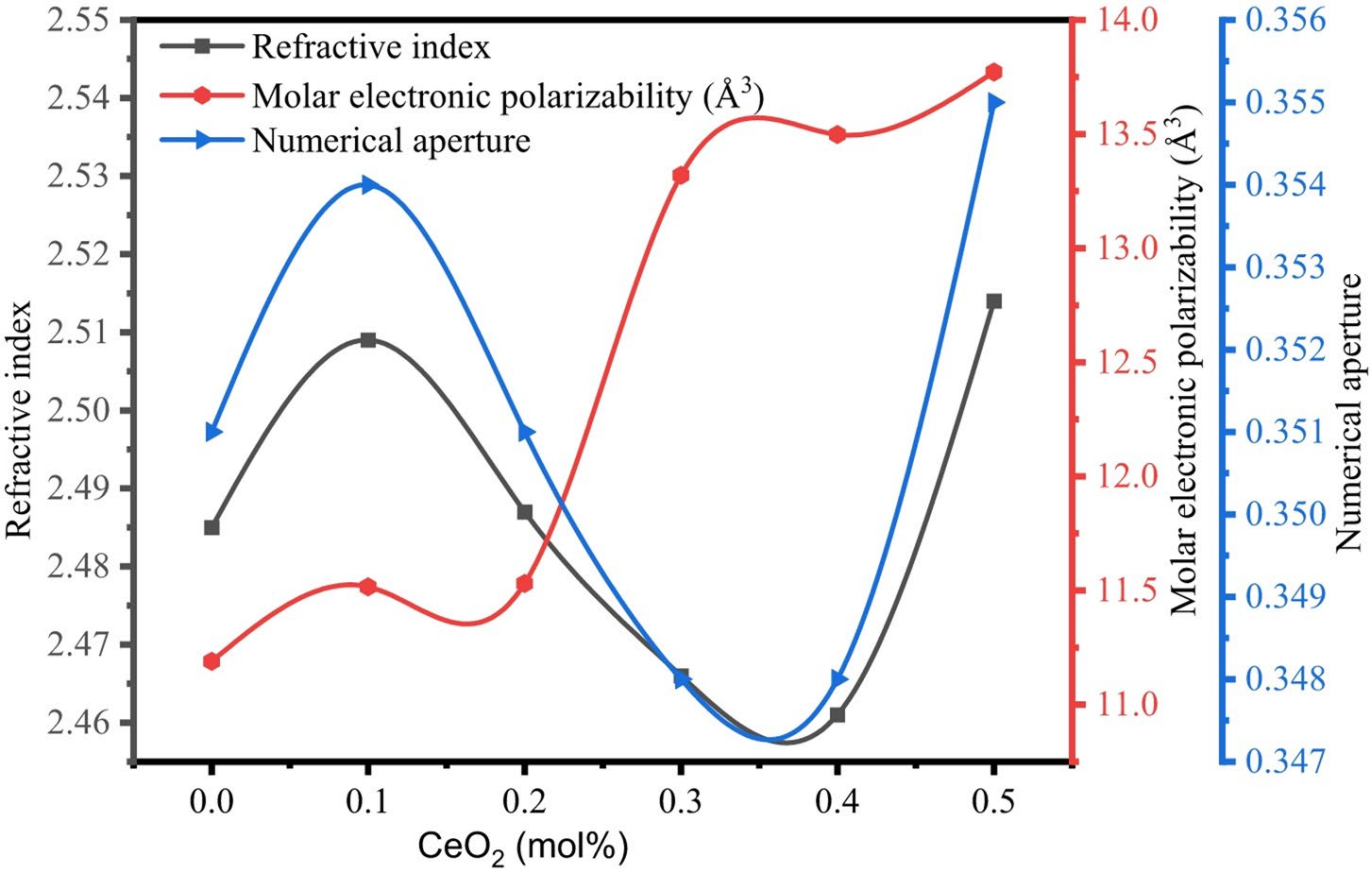
Variation in refractive index, molar electronic polarizability and numerical aperture of BTNC glasses.

Where $\:n$ is the RI of the glass, and Δ represents the fractional refractive index change (0.01)^38,39,70^. The BTNC glasses can be utilised as a core material in the manufacturing of optical fibre cables if the numerical aperture lies between 0.12 and 0.5. The NA varies between 0.348 and 0.355 in BTNC glasses, as shown in Table 5. Hence, these glasses are potential candidates in the manufacturing of optical fibre as a core material^70,71^. The correlation between the average refractive index, molar electronic polarizability and numerical aperture is shown in Fig. 14.

**Table 5:** Refractive index ($\:n$), Molar refraction ($\:{R}_{m}$) and Molar electronic polarizability ($\:{\alpha\:}_{m}$) with error ± 0.001.

Glass code	$\:{\boldsymbol{n}}_{\boldsymbol{D}-\boldsymbol{S}}$	$\:{\boldsymbol{n}}_{\boldsymbol{K}-\boldsymbol{S}}$	$\:{\boldsymbol{n}}_{\boldsymbol{H}-\boldsymbol{V}}$	$\:{\boldsymbol{n}}_{\boldsymbol{R}-\boldsymbol{S}}$	$\:{\boldsymbol{n}}_{\boldsymbol{a}\boldsymbol{v}\boldsymbol{g}}$	$\:{\boldsymbol{R}}_{\boldsymbol{m}}$ (cm^3^/mol)	$\:{\boldsymbol{\alpha\:}}_{\boldsymbol{m}}$ (Å^3^)	NA
BTNC0	2.426	2.603	2.380	2.528	2.485	28.198	11.190	0.351
BTNC1	2.448	2.627	2.406	2.557	2.509	29.021	11.516	0.354
BTNC2	2.428	2.606	2.383	2.532	2.487	29.058	11.531	0.351
BTNC3	2.408	2.585	2.361	2.507	2.466	33.563	13.319	0.348
BTNC4	2.404	2.581	2.502	2.356	2.461	34.014	13.498	0.348
BTNC5	2.453	2.631	2.562	2.410	2.514	34.706	13.772	0.355

#### Reflection loss ($\:{\boldsymbol{R}}_{\boldsymbol{L}}$), and metallization criterion ($\:\boldsymbol{M}$)

The reflection loss of the BTNC glasses was measured using a mathematical relation^41^:

28 $$\:{R}_{L}={\left(\frac{n-1}{n+1}\right)}^{2}$$

The quantity of Ce ions increases as the CeO_2_ concentration increases; however, the reflection loss is low. The $\:{R}_{L}$ values are mentioned in Table 6. The lower and nearly consistent values of R_L_ (17.8% − 18.6%) indicate that the glass host is capable of accommodating cerium ions without much optical loss^46,72^.

The refractive index and Lorentz-Lorentz equation have been used to examine the metallic and non-metallic nature of BTNC glasses. The Herzfeld theory of metallization^73^ states that the substance can be non-metal if R_m_/V_m_ is less than 1; otherwise, it can be metal if R_m_/V_m_ is greater than 1. The metallization criterion is calculated by using the Herzfeld theory of metallization. Additionally, there are other formulas available in literature, i.e., Dimitrov and Sakka proposed equations for the metallization criterion by using refractive index M_n_ and the metallization criterion by using energy gap M_g_. The obtained metallization values are in good agreement with each other^74^.

29 $$\:{M}_{H}=1-\frac{{R}_{m}}{{V}_{m}}$$

30 $$\:{M}_{g}=\sqrt{\frac{{E}_{g}}{20}}$$

31 $$\:{M}_{n}=1-\frac{{n}_{avg}^{2}-1}{{n}_{avg}^{2}+2}$$

M_avg_ values of BTNC glasses lie in the range of 0.336 to 0.347. The change in the $\:{M}_{H}$, $\:{M}_{g}$, $\:{M}_{n}$, and $\:{M}_{avg}$ values of BTNC glasses are reported in Table 6. Generally, higher M values, close to one, are attributed to an insulating nature, and lower M values, close to zero, are ascribed to a narrow band gap or conductors due to an enhanced metallic nature. The variation in M values because of band gap energy signifies that the glass samples are not metallizing; however, the width of the bands is relatively smaller^75^.

#### Dielectric constant ($\:\boldsymbol{\epsilon\:}$), Optical dielectric constant ($\:{\boldsymbol{\epsilon\:}}_{\boldsymbol{o}\boldsymbol{p}\boldsymbol{t}}$), and Linear dielectric susceptibility ($\:{\boldsymbol{\chi\:}}_{\boldsymbol{d}}$)

It is reported that in glasses, the dielectric constant ($\:\epsilon\:$), optical dielectric constant ($\:{\epsilon\:}_{opt}$), and linear dielectric susceptibility ($\:{\chi\:}_{d}$) depend not only on the$\:\:n$, but also on ionic and electronic polarizability^76^.

32 $$\:\epsilon\:={n}^{2}$$

33 $$\:{\epsilon\:}_{opt}=\epsilon\:\frac{dt}{dp}=\epsilon\:-1={n}^{2}-1$$

Here, p refers to polarisation.

34 $$\:{\chi\:}_{d}=\frac{\epsilon\:-1}{4\pi\:}$$

From Table 6, it is noticed that the dielectric constant and optical dielectric constant vary from 6.029 to 6.293 and 5.029 to 5.293, respectively, with increasing CeO_2_ concentration. The $\:{\chi\:}_{d}$ values vary between 0.421 and 0.400 and are tabulated in Table 6. These significant variations indicate that the addition of CeO_2_ can alter both extinction and optical absorption.

**Table 6:** Reflection loss ($\:{R}_{L}$), and Metallization Criterion (M), Dielectric constant ($\:\epsilon\:$), Optical dielectric constant ($\:{\epsilon\:}_{opt}$), and Linear dielectric susceptibility ($\:{\chi\:}_{d}$) with error ± 0.001.

Glass code	$\:{\boldsymbol{R}}_{\boldsymbol{L}}$	$\:{\boldsymbol{M}}_{\boldsymbol{H}}$	$\:{\boldsymbol{M}}_{\boldsymbol{g}}$	$\:{\boldsymbol{M}}_{\boldsymbol{n}}$	$\:{\boldsymbol{M}}_{\boldsymbol{a}\boldsymbol{v}\boldsymbol{g}}$	$\:\boldsymbol{\epsilon\:}$	$\:{\boldsymbol{\epsilon\:}}_{\boldsymbol{o}\boldsymbol{p}\boldsymbol{t}}$	$\:{\boldsymbol{\chi\:}}_{\boldsymbol{d}}$
BTNC0	0.182	0.280	0.381	0.368	0.343	6.146	5.146	0.410
BTNC1	0.185	0.275	0.375	0.363	0.338	6.270	5.270	0.420
BTNC2	0.182	0.279	0.380	0.368	0.342	6.160	5.160	0.411
BTNC3	0.179	0.284	0.385	0.372	0.347	6.054	5.054	0.402
BTNC4	0.178	0.285	0.386	0.374	0.348	6.029	5.029	0.400
BTNC5	0.186	0.274	0.374	0.362	0.337	6.293	5.293	0.421

#### Electronic oxide polarizability ($\:{\boldsymbol{\alpha\:}}_{{\boldsymbol{O}}^{2-}}$), Optical Basicity ($\:\boldsymbol{\varLambda\:}$), Electronegativity ($\:\boldsymbol{\chi\:}$), and Electric susceptibility ($\:{\boldsymbol{\chi\:}}_{\boldsymbol{e}}$)

**Fig. 15:**
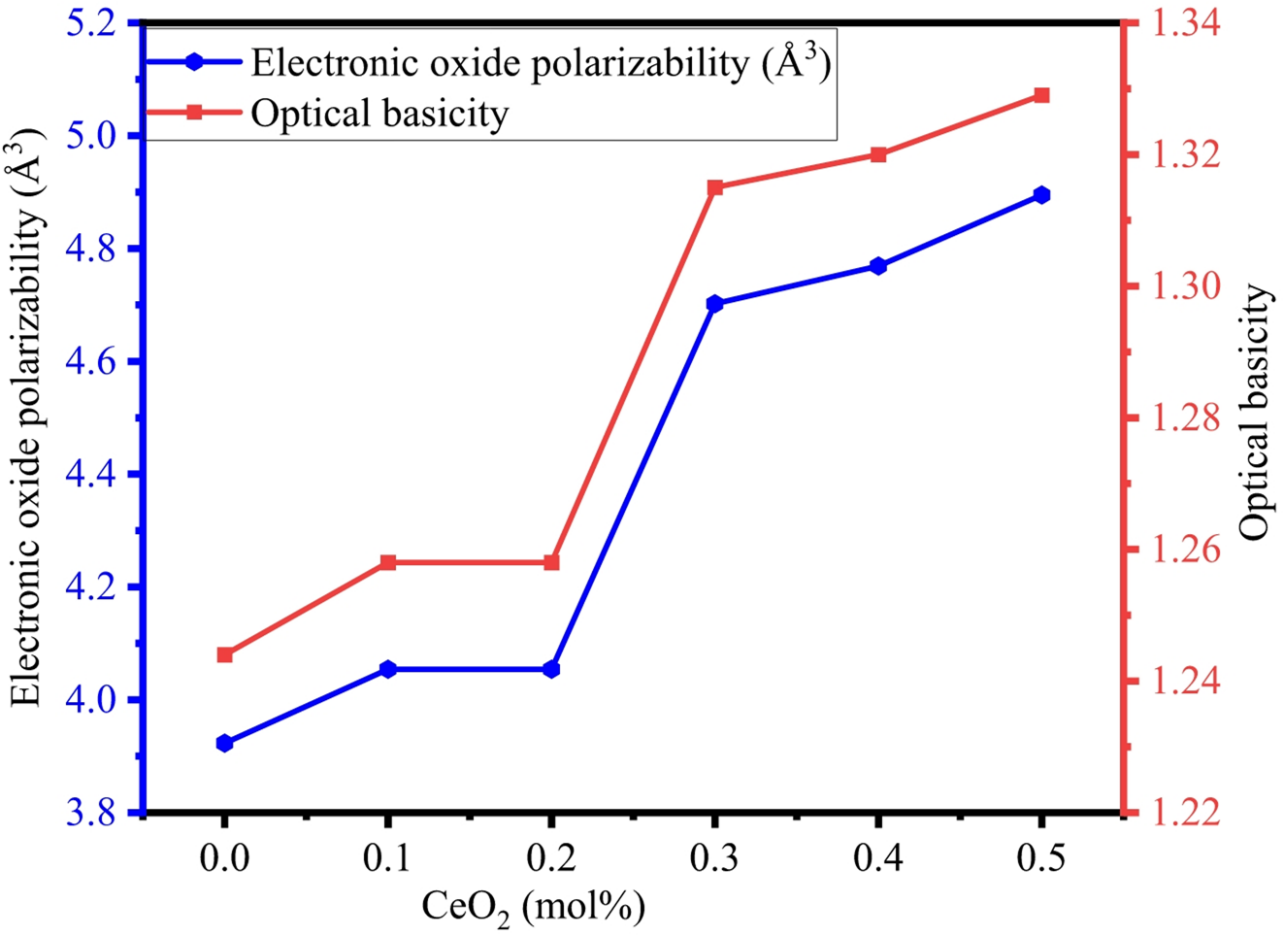
Variation of electronic oxide polarizability and optical basicity of BTNC glasses.

**Fig. 16:**
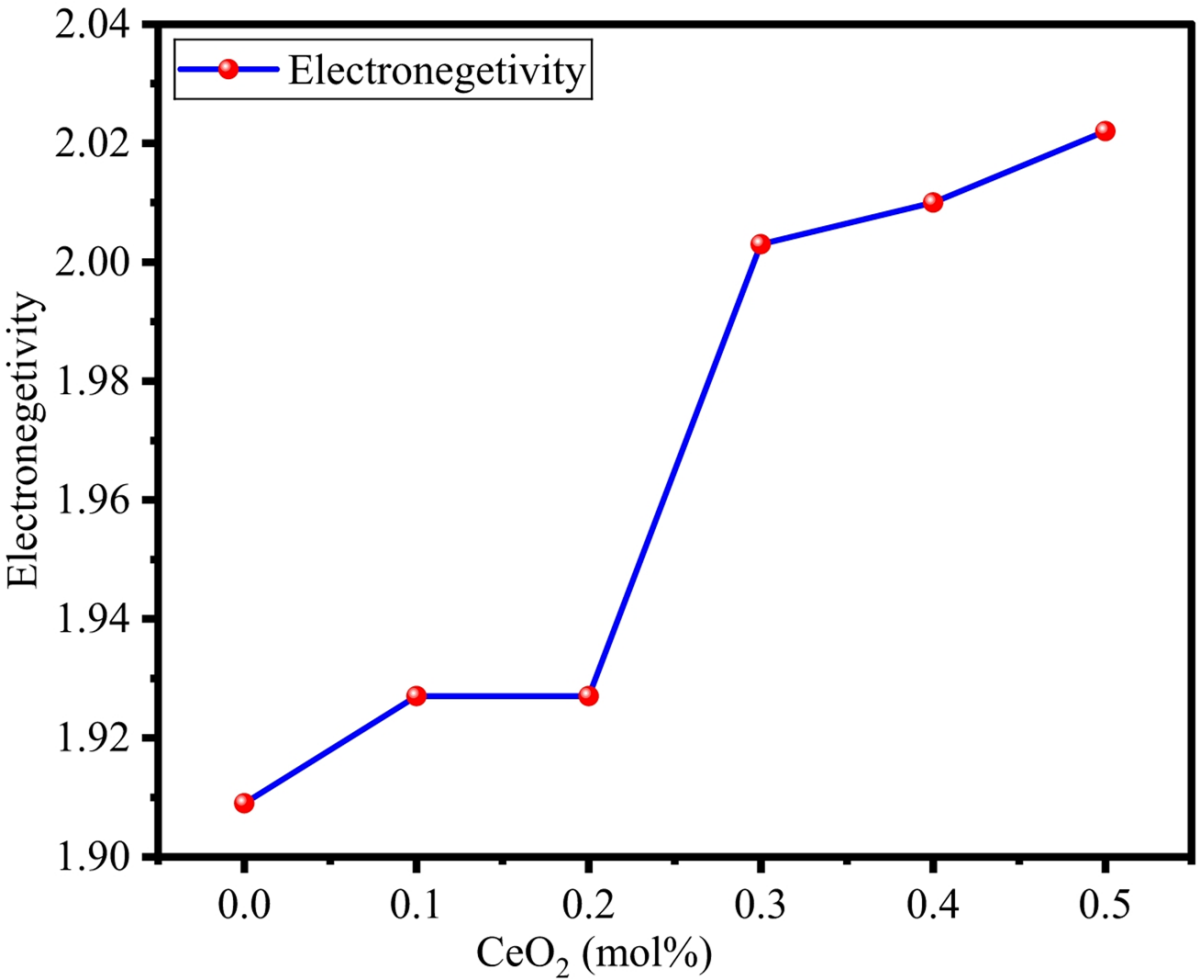
Variation of electronegativity with CeO_2_ of BTNC glasses.

The electronic polarizability of an electron cloud displays its ease of distortion when subjected to an external electric field. Equations 9 and 10, discussed in Sect. 2.3.2, were used to calculate the electronic oxide polarizability and optical basicity of the BTNC glasses using the average refractive index values. The results indicated an increase in optical basicity from 1.244 to 1.329 and electron oxide polarizability from 3.923 Å^3^ to 4.895 Å^3^. The polarizability of oxide ions and optical basicity exhibit an increasing pattern as illustrated in Fig. 15, and values are noted in Table 7. The addition of Ce ions with accessible 4f/5d orbitals increases electric distortion because of their inherent polarizability. Furthermore, CeO_2_ functions as an intermediate oxide, partially breaking the concentration of NBOs. The loosely bound electrons contribute to polarizability^69^. Equations 11 and 12, as discussed in Sect. 2.3.2, were used to compute the electronegativity and electric susceptibility of the BTNC glasses using optical basicity and the average refractive index values, respectively. Figure 16 displays the variation of electronegativity with respect to the CeO_2_ concentration of BTNC glasses. Electronegativity increased from 1.909 to 2.022, and electric susceptibility increased from 0.410 to 0.421. The calculated values are listed in Table 7. Increased electronegativity improves structural stability and bandgap management, enabling BTNC glasses towards advanced optoelectronic applications. Increased optical basicity will enhance luminescence efficiency, making the glasses favourable to advanced display applications^77,78^. The combined effects improve electronegativity and electric susceptibility, leading to an improved dielectric response suitable for photo-electronic device application^79^.

#### Third-order susceptibility ($\:{\boldsymbol{\chi\:}}^{\left(3\right)}$) and nonlinear refractive index ($\:{\boldsymbol{n}}_{2}$)

Third-order susceptibility and nonlinear refractive index behaviour are important because dielectric materials exhibit nonlinear features when it is subjected to high-intensity electromagnetic waves. $\:{\chi\:}^{\left(3\right)}$ and $\:{n}_{2}$ are calculated by using the Ticha-Tichy formula^80^ given below:

35 $$\:{\chi\:}^{\left(3\right)}=A{\left[\frac{{n}^{2}-1}{4\pi\:}\right]}^{4}$$

36 $$\:{n}_{2}=\frac{12\pi\:{\chi\:}^{\left(3\right)}}{n}$$

**Fig. 17:**
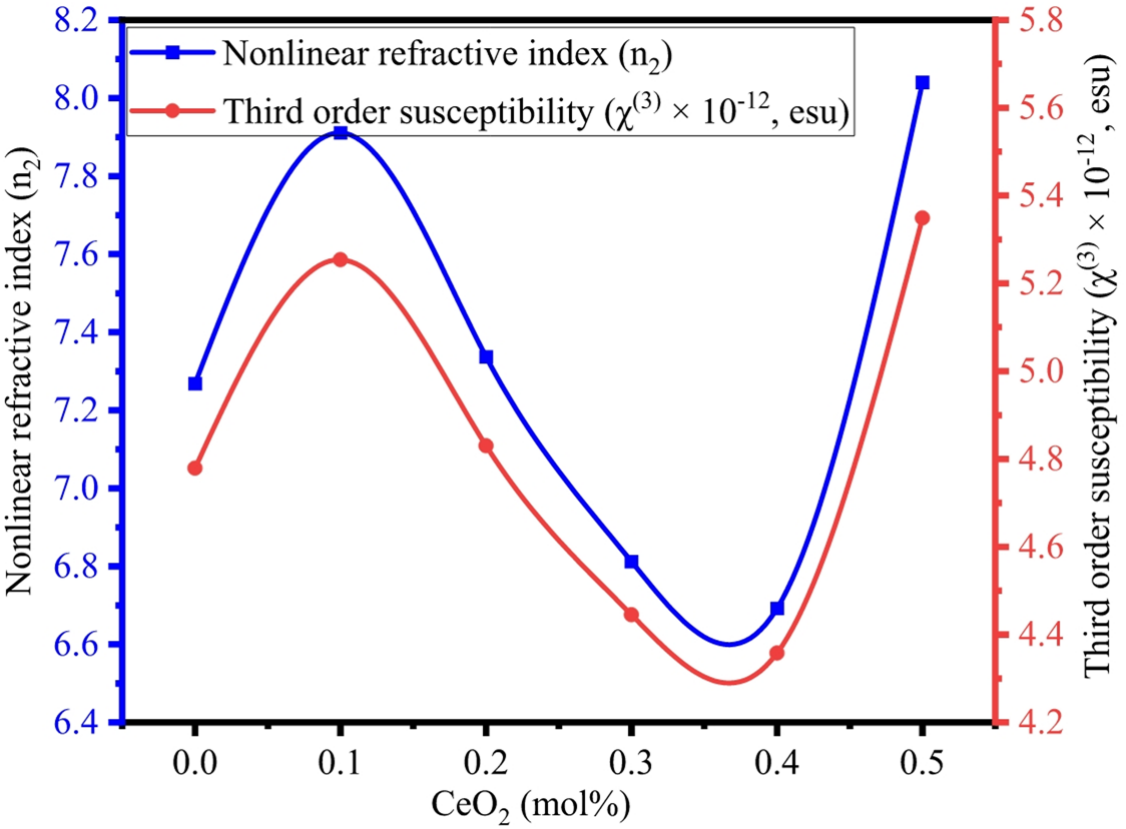
Variation of third-order susceptibility and nonlinear refractive index of BTNC glasses.

Where A is a constant (1.7 × 10^− 10^ esu). The inclusion of CeO_2_ in the BTNC glasses showed an increase in $\:{\chi\:}^{\left(3\right)}$ values from 4.799 × 10^− 12^ esu to 5.349 × 10^− 12^ esu and $\:{n}_{2}$ values from 7.268 × 10^− 11^ esu to 8.040 × 10^− 11^ esu correspondingly and are listed in Table 7. The incorporation of CeO_2_ generates NBOs with increased electronic oxide polarizability and susceptibility; this increase in linear optical characteristics corresponds with increased $\:{\chi\:}^{\left(3\right)}$ and $\:{n}_{2}$^77,81^. Figure 17 depicts the variation of third-order susceptibility and nonlinear refractive index of BTNC glasses. The increase in $\:{\chi\:}^{\left(3\right)}$ and $\:{n}_{2}$ values than other glasses^82^ shows that the materials are suitable for ultrafast photonic applications and optical switching devices.

**Table 7:** Electronic oxide polarizability ($\:{\alpha\:}_{{O}^{2-}}$), Optical Basicity ($\:\varLambda\:$), Electronegativity ($\:\chi\:$), Electric susceptibility ($\:{\chi\:}_{e}$), Third-order susceptibility ($\:{\chi\:}^{\left(3\right)}$) and Nonlinear refractive index ($\:{n}_{2}$) with error ± 0.001.

Glass code	$\:{\boldsymbol{\alpha\:}}_{{\boldsymbol{O}}^{2-}}$, (Å^3^)	$\:\boldsymbol{\varLambda\:}$	$\:\boldsymbol{\chi\:}$	$\:{\boldsymbol{\chi\:}}_{\boldsymbol{e}}$	$\:{\boldsymbol{\chi\:}}^{\left(3\right)}$ × 10^− 12^, esu	$\:{\boldsymbol{n}}_{2}$ × 10^− 12^, esu
BTNC0	3.923	1.244	1.909	0.410	4.779	7.268
BTNC1	4.054	1.258	1.927	0.420	5.254	7.911
BTNC2	4.054	1.258	1.927	0.411	4.830	7.337
BTNC3	4.702	1.315	2.003	0.402	4.445	6.812
BTNC4	4.769	1.320	2.010	0.400	4.358	6.692
BTNC5	4.895	1.329	2.022	0.421	5.349	8.040

### Photoluminescence studies

#### Excitation and emission spectra

**Fig. 18:**
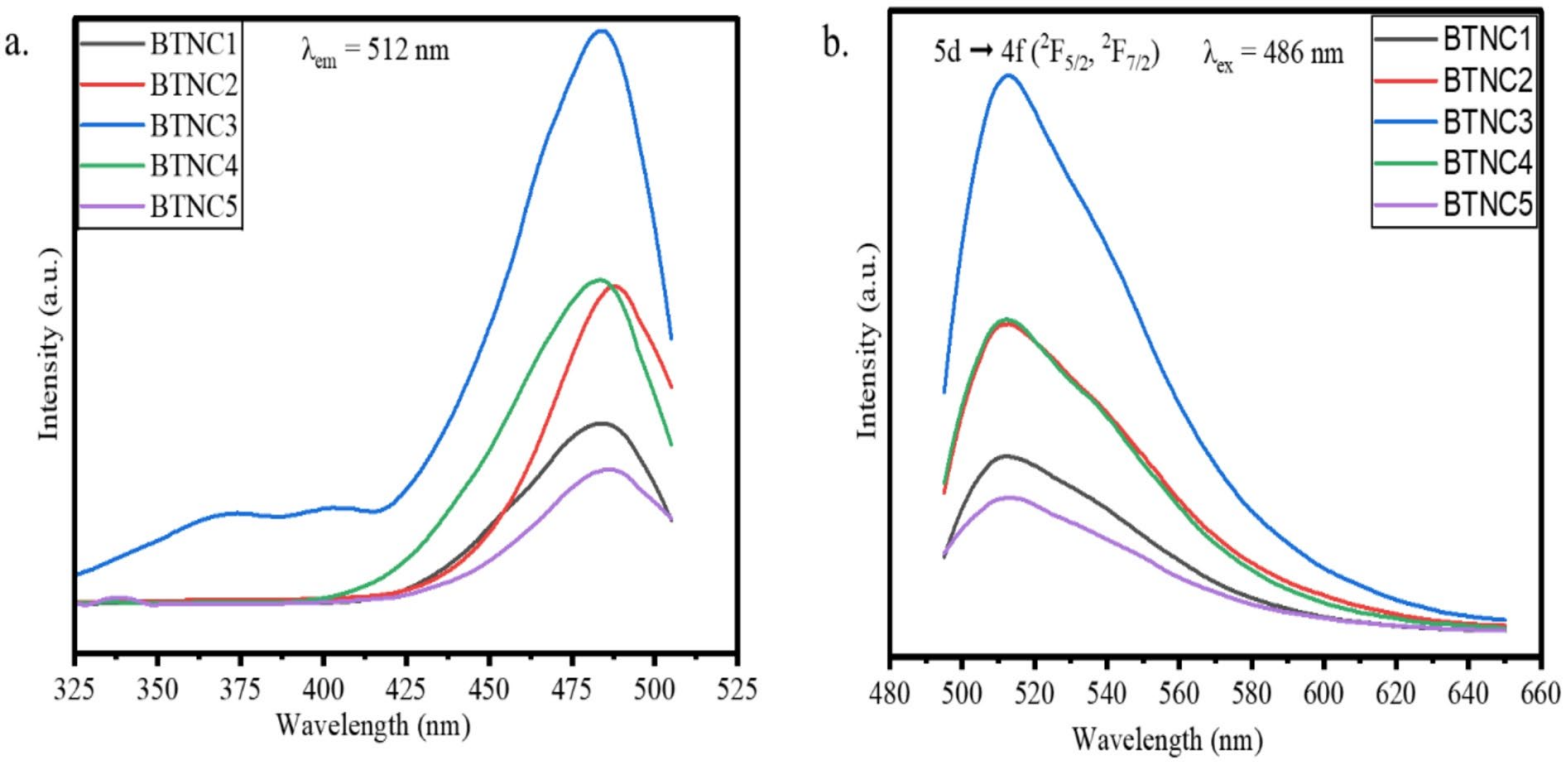
(**a**). Excitation spectra (**b**). Emission spectra of BTNC glasses doped with CeO_2_.

The excitation spectra of CeO_2_-doped BTNC glasses were obtained by choosing an emission wavelength of 512 nm and are shown in Fig. 18. (a) The photoluminescence spectra of the prepared CeO_2_-doped BTNC glasses were recorded between 490 nm and 650 nm based on the excitation spectra. The emission spectra depicted in Fig. 18. (b) The obtained emission spectra showed a broad band with a peak centred at 512 nm, which corresponds to green emission, and a band spanning the region 490–580 nm. The CeO_2_-doped BTNC glasses emit a wide green emission at 512 nm due to allowed 5 d → 4f (^2^F_5/2_, ^2^F_7/2_) transitions of Ce^3+^ ions. It has been observed that the intensity varies with CeO_2_ concentration, but no significant shift in wavelength. The intensity increases up to 0.3 mol% of CeO_2_ and then diminishes due to concentration quenching. The electron-phonon coupling and structural modification in the glass matrix have a significant impact on emission and the broadness of the band. The transition 5 d → ^2^F_5/2_ emits at 512 nm, and 5 d → ^2^F_7/2_ contributes to the broadening of the band. Since the surrounding environment highly influences the 5 d level, the emission appears as a broad green band rather than sharp lines^83–86^.

### CIE diagram and color correlated temperature (CCT)

**Fig. 19:**
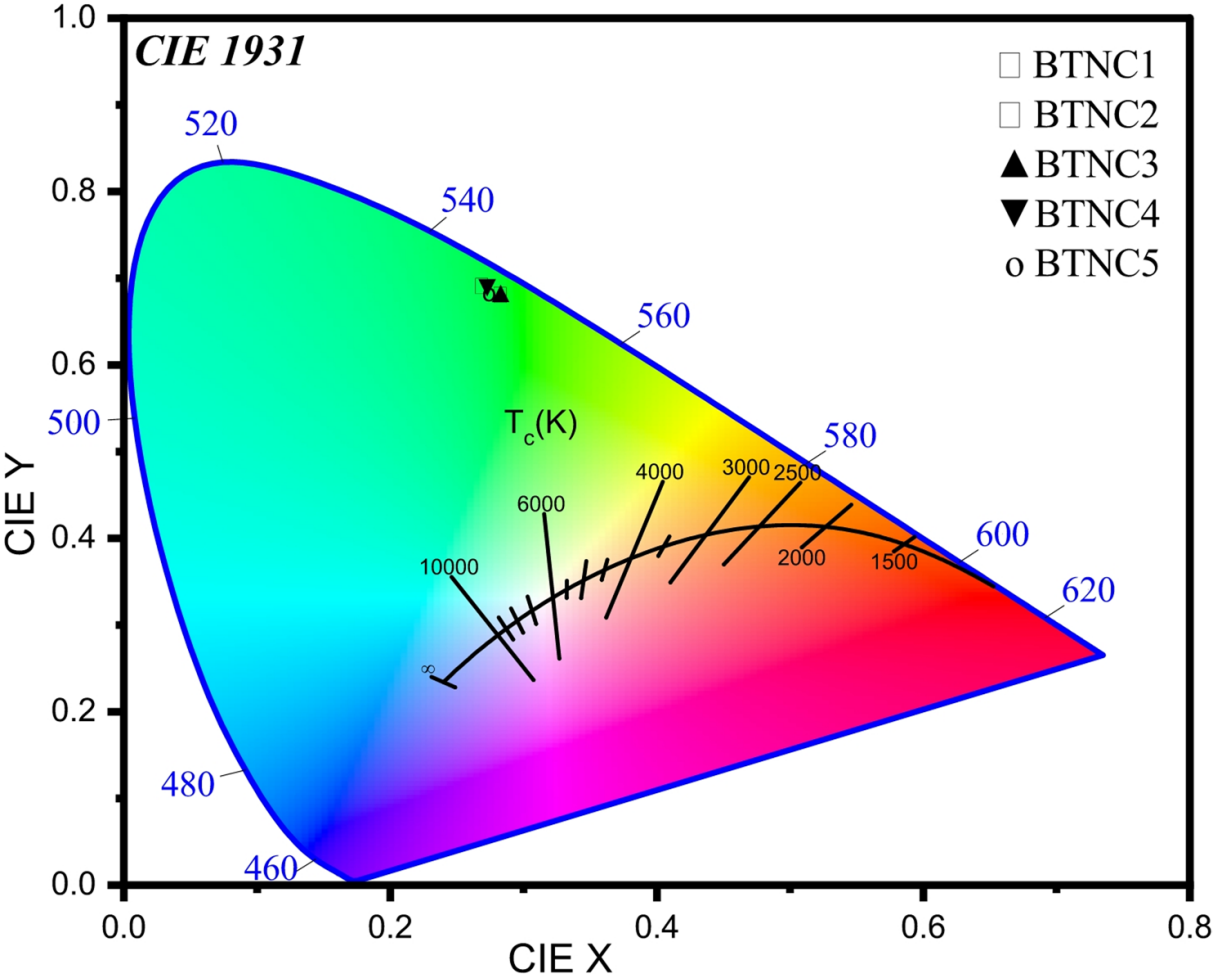
CIE diagram of BTNC glasses doped with cerium.

The closest distance between a light-source emission coordinates and the coordinates of the Planck emission spectrum is a crucial measurement for determining its ability to reproduce a natural light source. Therefore, to compute an approximation and validate the emission color, the CIE 1931 diagram is generated by using emission data and is shown in Fig. 19. The color chromaticity coordinates of the prepared glasses can be used to analyze the reliable luminescence color of Ce^3+^. The points corresponding to CeO_2_-doped BTNC glasses appeared in the green to yellowish region. This verifies the appearance of a broad band range of green to yellowish. So, the results indicate that the obtained glasses have potential applications in light-emitting devices^87^. The CCT is determined using (x, y) coordinates in the McCamy relation^88^.

37 $$\:CCT=\:-449{a}^{3}+3525{a}^{2}-6823a+5520.3$$

Where, $\:a\:=\:x\:-\frac{0.332}{y}-\:0.186,$ is the inverse line.

The determined (x, y) coordinates from CIE and CCT values of prepared BTNC glasses are tabulated in Table 8. The CCT values showed greater than 5000 K, indicating a cool CCT. According to the findings and evaluation, the BTNC glasses are more suitable for light-emitting devices.

**Table 8:** CIE coordinates and CCT values.

Sample Code	CIE Coordinates		CCT (K)
	x	y	
BTNC1	0.255	0.672	6691.6
BTNC2	0.269	0.662	6486.2
BTNC3	0.267	0.662	6518.9
BTNC4	0.257	0.669	6666.5
BTNC5	0.263	0.663	6582.4

## Conclusion

The CeO_2_-BTNC glasses were synthesized by the melt-quenching method. The amorphous state of BTNC glasses was confirmed through the XRD. The existence of functional groups BO_4_, BO_3_, TeO_3_, and TeO_4_, and B-O-B and Ce-O bonds was explored through the ATR-FTIR spectroscopic technique and deconvolution of the resulting spectra. The density varies from 3.347 g/cm^3^ to 2.746 g/cm^3^. The direct band gap varies from 2.896 eV to 2.801 eV. The average refractive index values of different theoretical approaches were found in the range of 2.461–2.514. The metallization criterion and numerical aperture are in the range of 0.337–0.386 and 0.348–0.355, respectively, which can be considered for optical devices. The electronic oxide polarizability and optical basicity were in the range of 3.923 ${{A}^3}$ – 4.895 $A^{3}$ and 1.244–1.329, respectively. The Third-order susceptibility and nonlinear refractive index values range from 4.799 × 10^− 12^ esu to 5.349 × 10^− 12^ esu and from 7.268 × 10^− 11^ esu to 8.040 × 10^− 11^ esu, respectively. The CeO_2_-doped BTNC glasses allowed 5 d → 4f (^2^F_5/2_, ^2^F_7/2_) transitions of Ce^3+^ ions and emitted a wide green emission at 512 nm. The CIE diagram showed that CeO_2_-doped BTNC glasses lie in the green to yellowish region. The CCT values are > 5000 K, indicating a cool CCT. The highest photoluminescence emission intensity was observed for BTNC3 glass. According to the findings and analysis, the BTNC3 glass is more suitable for optoelectronic devices, including cool greenish light-emitting devices.

## Data Availability

Data will be made available on reasonable request by corresponding author.
